# Induced Pluripotent (iPSC) and Mesenchymal (MSC) Stem Cells for In Vitro Disease Modeling and Regenerative Medicine

**DOI:** 10.3390/ijms26125617

**Published:** 2025-06-11

**Authors:** Egor Panferov, Maria Dodina, Vasiliy Reshetnikov, Anastasia Ryapolova, Roman Ivanov, Alexander Karabelsky, Ekaterina Minskaia

**Affiliations:** Translational Medicine Research Center, Sirius University of Science and Technology, Olympic Avenue, 1, 354340 Sochi, Russia

**Keywords:** iPSCs, MSCs, stem cells, disease modeling, regenerative medicine, inherited diseases

## Abstract

In vitro disease modeling can be used both for understanding the development of pathology and for screening various therapies, such as gene therapies. This approach decreases costs, shortens research timelines, reduces animal testing, and may be more accurate in replicating the disease phenotype compared to animal models. This review focuses on the two types of stem cells: induced pluripotent stem cells (iPSCs) and mesenchymal stem cells (MSCs), which can be used for this purpose. Special attention is given to the impact of the isolation source and the variable expression of certain phenotypic markers on the differentiation capacity of these cells. Both similarities and critical differences between iPSCs and MSCs, as well as the outcomes of past and ongoing clinical trials, are discussed in order to gain insight and understanding as to which of these two cell types can be more suitable for the particular biomedical application.

## 1. Introduction

Developing new drugs is a long, labor-intensive, and therefore expensive process. The average cost of a full drug development cycle varies significantly depending on its therapeutic focus, ranging from $314 million to $4.46 billion, with an average of approximately $879.3 million, of which $515.8 million is spent on unsuccessful versions of the drug that failed in clinical trials [1]. Moreover, on average, nine out of ten drugs that reach the clinical trial phase fail. Typically, the reason for this is either insufficient clinical efficacy of the drug or its excessive, unforeseen toxicity [2]. One possible approach towards increasing the proportion of successful clinical trials, and therefore reducing the overall development costs, is to use more clinically relevant models at the preclinical stage [3]. Identifying the inefficiency of a drug at an earlier stage will halt its development in time, thus minimizing costs and increasing the efficiency of translation into the clinical setting. Disease models are also invaluable in identifying key pathogenetic mechanisms in basic research [4].

In vivo models, typically small lab animals, are traditionally considered to be the most representative and have a number of advantages that are practically impossible to reproduce when using other approaches. Well-developed animal models, when used in an appropriate context and when their known limitations are taken into account [5], can provide researchers with a great breadth of data on the systemic pathophysiology of diseases while allowing a significant degree of control over the experimental conditions that are difficult to simulate otherwise, such as the dietary variables and other environmental factors or stressors [6]. Indeed, complex pathologies involving multiple bodily systems, such as endocrine or immune diseases [7,8], as well as pathologies that significantly affect behavior [9], are difficult to reproduce without resorting to the use of laboratory animals.

Crucially, animal models can often exhibit different phenotypic manifestations of the same mutations compared to humans. These inconsistencies can be broadly divided into three groups: differences in morphology, differences in physiology, and differences in molecular pathways.

Morphological differences between humans and animals are particularly evident in the brain. Despite sharing many biochemical and functional similarities, the difference in complexity is such that, for example, most treatment strategies for traumatic brain injury developed in animal models tend to fail clinical testing despite performing well in a comparatively simpler animal brain [10]. The rodent pulmonary system is also considerably different from the human one. In addition to reduced branching, rodent airway epithelia contain little or no goblet cells at all, smaller bronchial glands, and lack respiratory bronchioles, which, among other things, makes rodents a suboptimal choice for modeling diseases like chronic bronchitis or chronic obstructive pulmonary disease [11].

Discrepancies between humans and lab animals are also prevalent in electrophysiological studies. Electrocardiogram recordings of human and mouse hearts are radically different [12], and no widely used lab animal model has been shown to correctly recapitulate the quantitative and kinetic qualities of all ion currents present in human cardiomyocytes, with only rabbits being somewhat reminiscent in terms of channel function [13]. Another physiological difference between humans and mice is the mechanism of both the visceral and the subcutaneous fat deposition, giving rise to heterogeneous populations of adipose cells, which differ intrinsically both within and among species. The most notable of these species-specific differences are in the exact anatomic localization of fat deposits, in the expression of the so-called “browning” genes, responsible for the conversion of white adipose cells to brown adipose cells, as the trends in their expression are opposite in mice and humans, and in the differences in secretion and function of adipose-derived hormones, as well as in the manifestations of hypoxia-related pathogenetic factors [14].

Molecular pathways in model animals, in general, are relatively poorly covered by transcriptomic data and Gene Ontology annotations compared to humans. Nonetheless, the pathway transferability from mice to humans seems to be relatively high, at 95%, with pigs and rats following close by, at 87% and 85%, respectively. The less conserved pathways include certain aspects of metabolism (particularly the ascorbate, aldarate, cyanoamino acid, porphyrin, chlorophyll, and pyruvate metabolism, as well as drug and xenobiotic metabolism by cytochrome P450), the Notch signaling pathway, antigen processing and presentation, and taste transduction and phototransduction, among other things [15]. While relatively niche, these differences can still potentially change the outcome of the experiment in an animal model and thus must be given due consideration if the desired outcome is the eventual clinical translation.

These discrepancies can overlap, resulting in a markedly different phenotype, which can often hinder or even preclude the meaningful use of animal models in some specific cases. An example is the different organization of the retina in humans and mice, which is the most commonly used in vivo model of retinopathies. The mouse retina is both morphologically significantly thinner and simpler than the human retina, and also contains photoreceptors that co-express both types of opsins. In addition, many commonly used mouse strains are characterized by the presence of mutations that further affect the anatomy and physiology of the retina [16]. Due to these differences, mouse models are typically unable to represent the progression of many retinal diseases, such as diabetic retinopathy, in their entirety, often exhibiting a much milder phenotype than the one found in humans [17].

Thus, while the complexity of the animal models is not seen as lacking compared to human studies, the representativeness of a particular disease phenotype in animals needs to be carefully assessed before planning the experiment [18].

Additionally, lab animal use in disease modeling is inherently associated with many ethical issues, which remain a contentious topic of discussion, both within the academic community and among the wider public [19]. Finally, maintaining laboratory animals is labor-intensive and requires significant financial costs, which makes scaling up experiments difficult or even impossible. Many preclinical research guidelines for in vivo animal experiments emphasize the importance of a sample size being large enough to achieve adequate power [20], which might be difficult to achieve with lab animals. Thus, despite their many considerable advantages, the rationality of the use of animal in vivo models, at least as far as preclinical research is concerned, has been brought into question in recent years, with suggestions even being made to reevaluate the necessity of their still largely ubiquitous use in later stages of preclinical trials and basic research [21,22,23,24].

In contrast, in vitro disease models, which can range from simple adherent cell lines [25] to elaborate bioengineered systems designed to mimic the three-dimensional (3D) tissue as closely as possible [26], are typically comparatively cheaper and thus more scalable. The major drawback of these models is their limited biological accuracy—simple two-dimensional (2D) adherent cell cultures are unable to replicate the complex network of interactions of different cell types, both with each other and with the extracellular matrix (ECM) [27]. While such interactions can be simulated in vitro to a limited extent, as outlined in the latter part of this review, these complex models are not only significantly more difficult to produce and maintain, but are also notoriously difficult to standardize as a result [28], meaning the obtained data suffer from a lack of reproducibility. As a result, even the more complex in vitro models cannot yet fully replace the in vivo models, and both are typically used in conjunction when extensive characterization is required.

Stem cell-based in vitro models are especially promising since they circumvent a number of problems associated with primary cultures. Unlike primary cultures, the collection of material requires an uncomfortable and complex biopsy procedure [29], and for a number of tissues, such as the cerebral cortex [30,31] or retina [32,33], this is possible only from postmortem material; stem cell-derived models originate from more accessible material. iPSCs are obtained by reprogramming cells from a number of widely available tissues [34]. In contrast, MSCs, which are directly present in adult tissues of the body, despite being comparatively less accessible, can still be obtained in some cases by relatively gentle methods from by-products of various medical procedures, such as the umbilical cord or subcutaneous fat remaining after plastic surgery [35].

In addition, many primary cell types do not remain viable under culture conditions for long durations without extensive supplementation of the growth medium [36], which makes it difficult to obtain large volumes of material and may differ depending on the donor. The ability of both types of stem cells (stem cells) to divide allows obtaining the necessary volumes of biological material, all the way up to industrial production [37,38].

These same features of stem cells are of interest in the context of regenerative medicine. For example, stem cell differentiation products can not only be used to study the mechanisms of disease or the effects of drugs, but can also be directly used to replace damaged tissue components [39]. The creation of complex 3D tissue models also contributes to the extension of fundamental knowledge about organogenesis and, in the future, can be used to create artificial organs. Considering that even with the increase in the number of donors, the need for organs currently continues to grow, with the demand far outstripping supply [40], and thus, this problem, unfortunately, remains a highly relevant one.

Despite both types of stem cells being capable of directed differentiation under appropriate conditions, few papers have explicitly compared their capability of serving as a platform for in vitro disease modeling. Therefore, the goal of our study was both to highlight the recent advances in disease modeling with iPSCs and MSCs, focusing primarily on hereditary diseases, and to discuss and compare their relative strengths and weaknesses when used in this context.

## 2. Comparative Characterization of iPSC- and MSC-Derived Disease Models

### 2.1. iPSC-Derived Models

Pluripotent stem cells, which include both embryonic stem cells (ESCs) and iPSCs, are characterized by self-renewal, the ability to differentiate into any cell type except for the extraembryonic structures [41], and the expression of transcription factors Oct4, Sox2, and NANOG, and cell surface antigens SSEA-1, SSEA-3, SSEA-4, TRA-1-60, TRA-1-81, and GCTM2 [42].

The first attempts to use human pluripotent stem cells for induced differentiation were historically associated with ESCs. ESCs are isolated from the inner cell mass of the developing blastocyst [43], typically sourced from surplus embryos after assisted reproduction techniques [44]. ESCs can be propagated in culture and can differentiate into a variety of somatic cell types [45]. Due to their low availability and the associated ethical concerns [46], the use of human ESCs is largely limited despite their potential utility.

A breakthrough came in 2006, when a team of scientists led by Dr. Shinya Yamanaka succeeded in obtaining pluripotent stem cells by reprogramming human somatic cells using four transcription factors—Oct4, Klf4, Sox2, and c-Myc (often abbreviated as OKSM [47,48])—and later dubbed the Yamanaka factors. These cells were named iPSCs. iPSCs closely resemble ESCs in terms of the gene expression profile and the active signaling pathways, and as such, both types of cells are typically considered to be mostly equivalent [49,50,51], with the iPSCs having the benefit of being significantly more accessible.

The choice of starting material for reprogramming is usually determined by its availability. In most cases, fibroblasts are used, but more exotic options are also available, such as urine-derived renal tubular epithelial cells or even hair follicle keratocytes [34]. Delivery of transcription factors into cells can also be carried out in different ways. Viral transduction is the most common method, both individually for each factor and using polycistronic expression cassettes [52,53].

The primary effect of the reprogramming process is the silencing of the somatic cell-specific genes, followed by the opening of the chromatin through the remodeling of the epigenetic landscape [54], allowing the pluripotency genes to be expressed. The retention of the individual genomes both after reprogramming and after differentiation is a huge advantage, as it allows the modeling of inherited diseases—iPSCs are widely used for studying highly penetrant genetic abnormalities with substantial phenotypic effects [55]. This is also a great blessing for personalized medicine (it is, for example, known that the adverse drug reactions are highly dependent on the individual), and iPSC screening can help make them more precise [56]. Finally, in a clinical setting, this allows for autologous transplantation of iPSC-derived tissues, significantly reducing the risk of immune rejection [57].

It should be noted that iPSCs are inherently heterogeneous—in fact, over 50% of the genome-wide expression variability in different iPSC cell lines can be attributed to the retention of a donor-specific expression pattern [58], with the remainder arising due to the genomic instability caused by the rapid division, desynchronization of the cell cycle, incomplete or aberrant reprogramming [59,60], and even varying cell culture conditions [61,62]. As a result, the differentiation potential of iPSCs and the properties of differentiated cells can vary even within an individual culture. For example, iPSCs differentiated into cardiomyocytes often give rise to multiple distinct populations, which, based on their morphology, gene expression, and electrophysiological properties, are broadly equivalent to atrial, ventricular, and nodal/pacemaker cardiomyocyte subtypes [63].

One of the primary factors contributing to the iPSC heterogeneity is the incomplete loss of the epigenetic profile of the cells of origin by the reprogramming process [64], although the genome of the donor is still the dominating factor [65,66]. Particularly notable is the retention of differentially methylated regions, located primarily in CpG islands, which have no equivalent in ESCs and apparently result from incomplete cell reprogramming [67]. The exact reasons for this differentially methylated region retention are currently unclear—while it is known that during the first reprogramming step, the essential pioneer transcription factors (which include Oct4, Sox2, and Klf4) bind the methylated nucleotides in CpG islands in order to initialize the demethylation through interactions with the FOXA proteins, the exact mechanisms of both the demethylation itself and the transcription factors’ interaction with the DNA demethylating machinery remain elusive and, thus, difficult to control experimentally [68]. From the practical standpoint, this means that iPSCs from different tissue sources have varying differentiation potentials. For example, iPSCs derived from mesodermal sources differentiate into the retinal tissue with lower efficiency than their ectodermal-derived counterparts [69]. In fact, the best iPSC tissue source for retinal differentiation seems to be the retina itself. Intriguingly, iPSCs derived from retinal neurons with the lowest reprogramming efficiency, namely the mature rods and immature bipolar neurons, end up having the highest retinal differentiation efficiency—this is likely because they retain most of the epigenetic marks associated with the original tissue [70]. Furthermore, the reprogramming process itself can impact differentiation. It has recently been shown that iPSCs obtained from multiple different tissues and with different reprogramming approaches all share elevated, albeit variable, levels of the progesterone receptor (PR) expression. This seemingly occurs regardless of whether PR was expressed in the progenitor cells or not, but is rather predominantly dependent on the chosen reprogramming approach, with the highest expression levels observed in iPSCs generated using the episomal vectors. The PR has previously been shown to be necessary for the differentiation of ESCs, which explains its presence in iPSCs as well. This fact must be taken into account when modeling tissues inherently responsive to steroid hormones, such as the mammary glands [71].

The chosen cell culture methodology can also affect the properties of iPSCs—iPSCs in suspension cultures not only demonstrate increased proliferative activity and a higher level of expression of pluripotency markers compared to adherent cultures, but also have a more stable genotype, without duplications or deletions in the eight most susceptible regions of the genome [72]. It is also important to note that iPSC-derived 2D cell cultures are characterized by an immature fetal phenotype, likely due to the absence of 3D interactions with other cells and the extracellular matrix, which are necessary for the correct recapitulation of cellular function in the mature tissue and, consequently, of the associated phenotype [73]. For example, iPSC-derived neurons have been shown to exhibit a more mature phenotype when embedded within a Poly Ethylene Glycol (PEG) hydrogel than in a 2D culture [74]. Similarly, iPSC-derived cardiomyocytes show a metabolic phenotype, contractile properties, and a gene expression profile resembling those of neonatal cardiomyocytes when cultured under regular adherent culture conditions [75].

However, complex, 3D organ-like aggregates capable of limited self-organization, called organoids, can be obtained from iPSCs, as well [76]. Compared to 2D cultures, organoid models are able to mimic the original tissues to a much greater extent, but also have a number of significant drawbacks of their own. First of all, in addition to the already mentioned problems with the heterogeneity of iPSCs themselves, there is a problem with the reproducibility of individual organoids (sometimes, even organoids grown in the same well of a culture plate can differ significantly from each other, and this is even more evident between different experiments and laboratories [77]). This significantly reduces the reproducibility of data obtained using such models, even in comparison with already heterogeneous 2D cultures of iPSC-derived cells, which is especially critical in preclinical studies, as the phenotype of the cells is known to directly impact their growth kinetics [78]. Another limitation is the impossibility of modeling system pathologies. A typical organoid is comprised only of the cells of the tissue type towards which the differentiation is directed and does not include, for example, its own vasculature as well as cells of the immune system. This limits the extent to which multisystem pathologies can be modeled. While approaches based on tissue self-organization, which involve adding cells to a differentiating organoid that can grow into the circulatory system, do exist [79,80,81], these models, called assembloids, are even less reproducible.

In a clinical setting, organoids hold great potential as autologous transplants—it has been shown that, at least in laboratory animals, transplanted organoids are able to integrate into the tissue and restore its function, which is of particular interest in the case of patient-derived organoids with gene-corrected mutations [82]. For example, human iPSC-derived cerebral organoids implanted at the border of the infarct core and the peri-infarct zone of a mouse that had suffered a stroke integrate into the nervous system after several months to such an extent that they are able to partially restore the sensorimotor functions lost after the stroke, which cannot be achieved by transplanting individual cells [83]. Similarly, transplantation of large liver organoids, obtained by fusing several smaller ones, into mice leads to the gradual ingrowth of liver vessels and ultimately allows the effects of chemically induced liver fibrosis to be ameliorated [84]. The efficiency of organoid transplantation can be improved by using them in conjunction with biocompatible materials. Adding biocompatible conductive nanofibers to cardiac organoids significantly increases the efficiency of their engraftment into the tissue, and in a mouse model of myocardial infarction, it restores cardiac function faster than in the case of similar organoids that did not contain these conductive elements [85].

**Figure 1:**
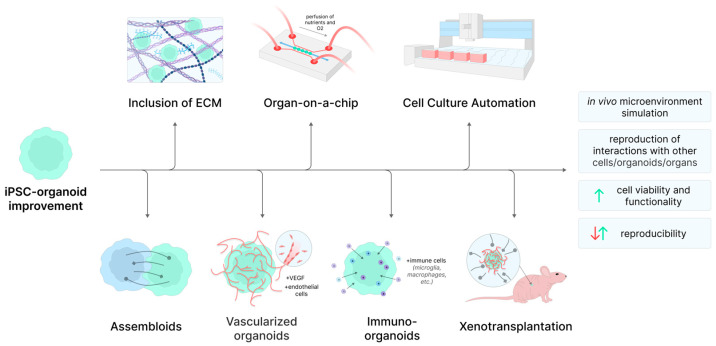
Different approaches towards improving iPSC-derived organoids. Organoid viability and maturity can be increased by the addition of the ECM to the culture system, culturing organoids on microfluidic chips (the organ-on-a-chip technology), or by the addition of vascular and immune cells to the organoid. Complex inter-tissue interactions can also be modeled by merging organoids with different tissues (including other organoids) into assembloids or by transplanting them into lab animals. All of these approaches result in decreased reproducibility of organoid models. It can, however, be increased by the automation of cell culture processes.

A number of existing problems of organoid models are currently being solved using advanced bioengineering methodologies, summarized in Figure 1. Controlled deposition of an iPSC mass using bioprinting allows not only to increase the reproducibility of experiments but also, in some cases, to increase their biological relevance [86]. The inability to reproduce system pathologies can be partially compensated for by using microfluidic systems. These organs-on-a-chip both allow the modeling of tissues characterized by a thorough flow of fluid, such as, for example, the intestines and lungs, and can even be used to combine several organoids into a single system, ensuring the exchange of metabolites, which enables the modeling of multi-organ pathologies [87].

To summarize, iPSCs are currently unparalleled in terms of the versatility of available differentiation strategies and allow for the creation of the most representative in vitro disease models. However, they are also expensive and challenging to work with, demanding a great deal of experience from the researcher, especially if reproducible results are desired, which is particularly important in rigorous preclinical research.

### 2.2. MSC-Derived Models

MSCs are a heterogeneous group of multipotent adult stem cells present in different tissues. According to the formal criteria introduced by the International Society for Cell and Gene Therapy (ISCT^®^) [88], all MSCs are characterized by at least the following:

- The ability to attach to plastic under cell culture conditions;
- Expression in >95% of the cell population of CD105, CD73, and CD90, but not CD45, CD34, CD14, CD11b, CD79a, CD19, or HLA class II;
- Osteogenic, chondrogenic, and adipogenic differentiation in vitro.

Moreover, the term MSC is used primarily in relation to cells that have the ability to self-renew and differentiate and are obtained after fractionation and selection from the total population of stromal cells expressing similar markers [89]. Despite the fact that experimental work with MSCs has been ongoing for many years, there is still no generally accepted consensus regarding either the nature of MSCs or the reporting guidelines—only 18% of published studies directly refer to the above ISCT criteria, and only 20% contain at least one functional assay confirming the differentiation capacity of the isolated cells [90]. Incomplete characterization of the MSC populations used in many experimental studies complicates the interpretation of accumulated data and leads to problems with the reproducibility of the results obtained.

Unlike iPSCs, where the isolation of material for reprogramming is usually accompanied by minimal invasiveness, the isolation of MSCs requires either invasive procedures or work with the medical waste left after such procedures. MSCs can be isolated from a variety of tissues, such as the bone marrow (BM-MSCs), umbilical cord blood (UC-MSCs), Wharton’s jelly (WJ-MSCs), adipose tissue (ASCs), dental pulp (DPSCs), the synovial membrane and fluid, peripheral blood, and even the placenta and amniotic membrane (AM-MSCs) [35,91]. There is a hypothesis that such a wide distribution of MSCs in various tissues of the body is due to their originating from pericytes located in the walls of capillaries [92]. It is proposed that only specific populations of pericytes act as precursors of MSCs—in response to certain external stressors, such as tissue injury, they are activated and produce MSCs, which then serve as mediators of tissue regeneration [93]. Indeed, cultured pericytes express a set of markers similar to MSCs [94]; however, they differ from them in the expression of some pluripotency genes and do not lose proliferative activity over time [95]. For example, pericytes, but not MSCs, are characterized by a high level of expression of the EBF1 transcription factor, which plays an important role in osteogenic and adipogenic differentiation, as well as an elevated expression of CD146 and a low level of CD90. Knockout of EBF1 leads to a decrease in the expression of CD146 and an increase in CD90, which corresponds to the ratio of these factors in MSCs [96].

As with the iPSCs, MSCs are characterized by a high degree of heterogeneity, which can be broadly divided into three levels: the donor, source tissue, and subpopulation within the tissue [97]. It has been shown that significant heterogeneity is characteristic of MSCs from the same tissue isolated from different donors. Factors such as age, body mass index, sex, and diseases play a role in this variability and manifest as differences in phenotype, gene expression and differentiation potential, division kinetics, and colony-forming capacity [98].

The source tissue also has a significant impact on the properties of the obtained MSCs. These include proliferative and migratory activity, clonogenicity, secretome composition, and, finally, differentiation potential [99,100]. MSCs are deeply integrated into their microenvironment, which includes, among other things, factors such as the substrate and ECM cues, pH, O_2_ tension, cytokines, hormones, and mechanical forces, all of which can change dramatically in different tissues, explaining the differences in phenotype observed in different MSC populations, including their differentiation potential [101,102]. For example, ASCs and BM-MSCs have the greatest potential to differentiate into hepatocytes; WJ-MSCs are capable of differentiating into dopaminergic neurons [103], while ASCs are noted as having the highest capability for osteogenic differentiation [104].

Finally, experimental data from recent years, often obtained using single-cell analysis methods, show that heterogeneity exists even within individual MSC niches, in which distinct populations can be identified [105]. For example, it is assumed that at least four distinct MSC subpopulations reside within the bone marrow, with different subpopulations having a preferentially osteogenic, chondrogenic, or adipogenic differentiation trajectory, and the terminal-stage quiescent cells comprising the fourth and final population [106]. Meanwhile, a different study has identified five distinct populations instead, shared between the WJ-MSC and BM-MSC. According to this study, the initial stem-like active proliferative cells first differentiate into multipotent progenitor cells, which can then become either unipotent preadipocytes or bipotent prechondro-osteoblasts, with the latter being capable of differentiation into unipotent prechondrocytes [107]. Another single-cell analysis shows that WJ-MSCs can also be divided into CD142-positive and -negative subpopulations, with the former performing better in a wound-healing assay [108]. Of course, the heterogeneity of MSCs affects their practical application and must be taken into account when planning an experiment or in their therapeutic use. Despite the large volume of data accumulated on MSC heterogeneity, the absence of well-established reporting guidelines makes comparisons and interpretations difficult [109].

As stated above, one of the main criteria defining MSCs is the ability to differentiate in three directions: osteogenic, adipogenic, and chondrogenic. In addition, the multipotent nature of MSCs under certain conditions also allows them to differentiate into some other tissues (different from the main three), although this property is rarely used for in vitro modeling, as outlined in the latter part of this review. It is also worth noting that, in contrast with typical fetal iPSC-derived cell cultures, MSC-derived osteoblast, chondrocyte, and adipocyte cultures typically possess a mature phenotype [110,111,112], although this is not necessarily true for other differentiation pathways—ASC- and UC-MSC-derived neural cells still exhibit an immature phenotype [113].

MSCs are typically maintained as 2D cultures, although 3D cultures have occasionally been reported, as well. 3D spheroid microenvironments result in drastic changes in MSC metabolism and gene expression profiles, resulting in improved differentiation potential and higher survival rates on the one hand, and heightened potential for epithelial–mesenchymal transitions on the other [114]. This makes them a promising tool for in vitro disease modeling and less so as a tool of regenerative medicine, given their potential to promote metastasis development in vivo. For example, culturing DPSC as a 3D floating sphere was shown to greatly prolong the expression of stem and even neural crest marker genes [115]. MSCs can be used to produce organoid models, as well, although this approach is significantly less common than organoid derivation from iPSCs. For example, culturing synovial MSCs in agarose microwells results in their self-aggregation into a spheroid by week 4, which can then be induced to differentiate into a cartilage organoid by the addition of chondrogenic medium containing dexamethasone, proline, insulin, transferrin, selenious acid, and GDF5. The resulting organoid can serve both as an in vitro model and as a therapeutic implement, as demonstrated by its transplantation into the mouse model of osteoarthritis, where it successfully mediated cartilage regeneration. Furthermore, vascularization of MSC organoids has been reported, as well. Culturing MSC-derived adipocytes with microvessel fragments isolated from adipose tissue in a 3D collagen matrix results in sprouting angiogenesis, giving rise to a vascularized construct reminiscent of the in vivo state of the tissue [116].

Unlike iPSCs, which are typically not directly injected into donors without any prior modification as part of the regenerative strategies (in fact, the injection of undifferentiated iPSCs into lab animals, which leads to the formation of teratomas, is one of the most reliable ways of proving their pluripotency experimentally [117]), certain proposed MSC therapy strategies are based on the assumption that they will differentiate into the components of the target tissue once injected into the site of injury. Transdifferentiation of MSCs into the tissue of interest can also be performed before transplantation in vitro [118]. For example, it has been shown that neural progenitor cells derived from ASCs are capable of supporting remyelination once injected into the brains of mice [119].

However, in recent years, it has become apparent that most therapeutic effects of MSCs, when injected into the site of injury, are driven not by replacing lost cells with their derivatives directly but rather by mobilizing the other resources of the tissue to stimulate the process of regeneration, as reviewed in [120,121], which led some researchers to call for MSCs to be reclassified as medicinal signaling cells [122]. Much like the differentiation potential, the MSC secretome also differs in composition depending on the source of isolation, although regardless of the source, it seems to have the common properties of regulating cell migration and homeostasis, as well as promoting cellular development and proliferation while inhibiting apoptosis [123,124].

Overall, MSCs’ differentiation capabilities seem to be of comparatively limited interest to researchers, with more attention currently being dedicated to the use of MSCs as a therapeutic vector rather than as a platform for in vitro disease modeling. Still, despite their reduced differentiation potential compared to iPSCs, MSCs possess a significant advantage of not requiring reprogramming to enter a state of stemness and are easier to maintain in culture.

## 3. Specific Examples of In Vitro Disease Modeling with iPSCs and MSCs

**Figure 2:**
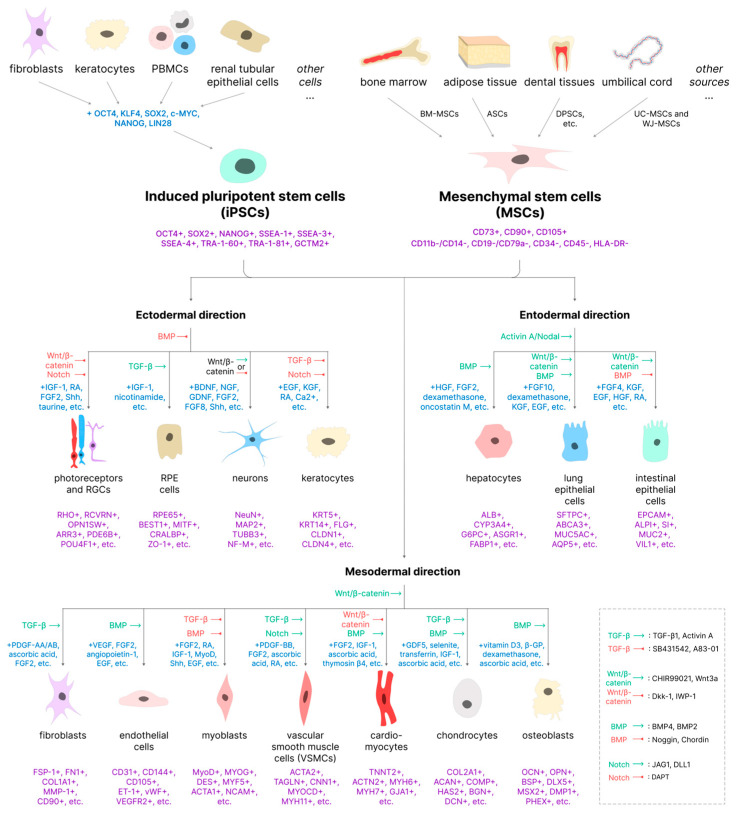
Derivation of iPSCs and MSCs from different sources and some of the main differentiation pathways. iPSCs and MSCs can be differentiated into the cells of ectodermal, mesodermal, and endodermal lineages, with varying efficiencies. The differentiation pathways and phenotypic markers shown in the figure are given for illustrative purposes only and are not an exhaustive list. Green arrows denote the signaling pathways that are activated in a particular differentiation pathway, while the red arrows show the pathways that are inhibited.

In this part of the review, we describe the particular advances in the in vitro modeling and regenerative therapy of hereditary diseases made in recent years. We do not aim to provide a comprehensive characterization of all existing strategies to model and treat these pathologies, which are briefly summarized in Figure 2, but rather to highlight certain trends and innovative approaches. We first briefly describe the pathological basis of the disease and then provide examples of how it might be modeled and treated with iPSCs and then with MSCs, drawing special attention to the differences between the two.

### 3.1. Ectodermal Derivatives

#### 3.1.1. Inherited Retinal Diseases

Inherited retinal diseases (IRDs) encompass a group of rare genetically and phenotypically heterogeneous conditions caused by mutations in over 300 genes [125]. They are most commonly associated with dysfunction and degeneration of the retinal pigment epithelium (RPE) cells, photoreceptor cells, and retinal ganglion cells (RGCs), all of which play essential roles in retinal visual function. As a result, patients experience progressive vision loss, often leading to complete blindness at a relatively young age. This group of diseases includes retinitis pigmentosa (RP) [126], Leber congenital amaurosis (LCA) [127], Stargardt disease (SD) [128], and others. RP is the most common IRD and is associated with more than 90 genes, with mutations in the *RHO*, *PRPF31*, *USH2A*, and *RPGR* genes being the most frequent causes [129]. Furthermore, there are over 80 syndromic forms of RP [130]. Among the most well-known are Usher syndrome and Bardet–Biedl syndrome, which are characterized by the presence of concomitant conditions, such as deafness, polycystic kidney disease, etc. [131]. Age-related macular degeneration (AMD), a complex, multifactorial disease primarily associated with aging, is also characterized by genetic predisposition [132].

However, in most cases, IRDs are monogenic diseases, which, together with the immune-privileged status of the eye as an organ [133] and the accessibility of the retina for surgical intervention, makes them especially suitable for treatment with gene therapy methods [134,135]. For example, in 2017, the FDA approved gene therapy for type 2 LCA using the adeno-associated virus (AAV)-based drug Luxturna, which delivers a functional copy of the *RPE65* gene to retinal cells [136]. Currently, the development of new drugs for the treatment of IRDs requires the use of relevant in vitro models at the preclinical stage, since confirmation of efficacy only in in vivo models, which often do not reproduce the phenotypic complexity of human retinal diseases, may not be sufficient to successfully complete clinical trials [137]. At the moment, one of the promising areas is the development of in vitro models of retinal diseases based on stem cells of various origins [16,138].

Both MSCs and iPSCs are capable of 2D differentiation into retinal cells, resulting in a heterogeneous population of cells differentiated with varying efficiency. Detailed differentiation protocols for MSCs and iPSCs are provided in numerous review articles [139,140]. Currently, 2D differentiation protocols allow obtaining functional RPEs from iPSCs with a yield of up to 90% after sorting [141]. In comparison, the efficiency of differentiation into photoreceptors and RGCs is noted as being much lower and less stable, reaching 30–40% at most [142]. Moreover, the yield and maturity of retinal cells derived from MSCs are even lower [140].

The generation of mature photoreceptors with outer segments (OS), which are important for phototransduction due to the light-sensitive opsins they contain, has become possible from 3D retinal organoids (ROs) derived from iPSCs. ROs include the major retinal cell types arranged in a manner consistent with the layered structure of the retina in vivo. Methods for obtaining ROs have been described in recent review articles [143].

Although RGCs are among the first to develop from retinal progenitor cells on day 50 of differentiation, their numbers decline significantly as the organoid matures, beginning on day 90. This may be due to the lack of terminal synaptic connections to brain centers and inadequate cellular nutrition. In an attempt to address one of these issues, day 50 ROs were organized into an assembly model with cortical and thalamic organoids, resulting in RGCs extending axons deep into the assemblies and exhibiting increased survival and proliferation on day 150 [144]. Other disadvantages of ROs include the formation of RPE cells in a cluster that is not tightly adjacent to the photoreceptors, which contributes to the formation of photoreceptors with incorrect packing and orientation of membrane discs in OS-like structures. RO also lacks blood vessels and retinal immune cells of mesodermal origin, i.e., microglia [145]. In this regard, they can be improved using assembly and organ-on-a-chip technologies. For example, ROs cultured within a microfluidic platform in a hyaluronic acid-based hydrogel on top of a layer of iPSC-derived RPE cells contain three times as many OS-like photoreceptor structures with a mature phenotype as ROs grown under regular conditions. Furthermore, phagocytosis of OS by RPE cells was also observed in this model [146]. In a later study, iPSC-derived ROs and RPE cells from patients with the *USH2A* c.8559–2A>G RP-causing mutation were similarly cultured in Matrigel under perfusion conditions. It was noted that fewer photoreceptors in the ROs underwent apoptosis on the perfused chip, and the expression levels of specific markers were higher in RPE cells [147]. In addition, there are several studies modeling the outer blood–retinal barrier: the interaction between iPSC-derived RPE cells and the retinal choroid was mimicked by culturing RPE cells in hydrogel scaffolds; in one case with primary human vascular endothelial cells and fibroblasts [148], and in another, with iPSC-derived endothelial cells and MSCs [149]. In the latter study, iPSCs were derived from patients with Sorsby retinal dystrophy to model the disease phenotype of AMD and other macular degenerations and to study the independent role of RPE cells and their secreted factors. Recently, an attempt was made to integrate vascular structure into the ROs by culturing them with vascular endothelial cells and pericytes from iPSC-derived vascular organoids. The resulting vascularized retinal organoids responded to severe forms of diabetic retinopathy by reducing the size and number of RGCs [150]. Co-culture of ROs with iPSC-derived microglia-like cells or macrophage progenitor cells [151,152,153] has also been reported, leading to migration of these cells into the organoids and improved maturation of the latter.

Thus, significant advances have been made in replicating some of the in vivo conditions for the formation of more mature and functional ROs, but organoids that overcome all of these shortcomings have not yet been reported. The absence of this complex environment has so far allowed the production of ROs that are transcriptionally and functionally reminiscent of fetal retina tissues and are presumably developmentally restricted to 38 weeks [144]. This may pose challenges in modeling late-onset IRD, the pathogenesis of which is often associated with the accumulation of damage over time and age-related changes in the retina.

iPSCs are widely used to model various IRDs because they preserve the patient genotype, and the resulting RPE cells and ROs can recapitulate the genotype-phenotypic features of the disease and can be used to search for variants associated with it. Recent review articles have described numerous iPSC-based in vitro models for RP, LCA, SD, choroideremia, Best’s vitelliform dystrophy, AMD, and others [142,154]. For example, in one recent study, photoreceptor progenitors and ROs were generated from iPSCs from a patient with autosomal dominant RP caused by a mutation in the *RHO* gene c.644C>T, which showed incorrect localization of the rhodopsin protein, increased levels of autophagy markers, and endoplasmic reticulum stress [155]. In another study, ROs were generated from iPSCs from patients with monoallelic unresolved late-onset SD caused by mutations in the *ABCA4* gene. During organoid development, a disruption in photoreceptor localization was observed that correlated with disease severity, and activation of stress-related processes was detected in photoreceptors using RNA sequencing. Moreover, the authors were able to identify unspecified alleles and confirm functional splicing defects in ROs [156].

In addition to studying the pathogenesis of IRDs, iPSC-based models are also used to test new treatments for retinal diseases [142,157]. For example, a recent study demonstrated the efficacy of gene replacement therapy for type 4 LCA associated with mutations in the *AIPL1* gene. ROs derived from patient-derived LCA4 cells and cells with the *AIPL1* gene knockout using CRISPR/Cas9 were transduced with the AAV7m8.hRKp.AIPL1 virus, resulting in a sustained restoration of AIPL1, PDE6α, and PDE6β levels in photoreceptor cells and a decrease in cyclic guanosine monophosphate (cGMP) levels for up to 70 days [158]. In another study, iPSC-derived ROs and RPEs were used to optimize dual AAV split-intein adenine base editing (ABE) therapy used to correct the most common mutation in the *ABCA4* gene, c.5882G>A. Ultimately, the improved candidate demonstrated high levels of efficacy in mice and non-human primates in vivo, editing up to 75% of cone photoreceptors and 87% of RPE cells, making it promising for potential clinical applications [159]. In addition, in vitro iPSC-based models allow the testing of small molecules as therapeutic agents: for example, eupatilin and fasudil were recently tested on ROs derived from *LCA5* knockout iPSCs, which helped restore the organoid phenotype by reducing CEP290 and IFT88 accumulation in photoreceptor cilia and improving rhodopsin trafficking to the OS [160]. Attempts have also been made to automate the processes of creating iPSC-based models and screening therapeutic agents on them. For example, TECAN Fluent automated workstations were used to culture iPSCs from AMD donors, differentiate them into RPE cells, and perform the high-throughput screening of mitochondrial metabolism-affecting compounds [161]. The production of iPSCs and ROs using the Cell X robotic cell culture platform [162] and the robotic cell processing facility (R-CPF) incorporating the Maholo LabDroid universal humanoid robot [163] has also been reported.

Patient-derived iPSCs can also be used to generate retinal cells for cell replacement therapy. Dissociated photoreceptor and RGC cells, RPE sheets, or retinal layers are used for transplantation, and are sometimes combined with biodegradable scaffolds to improve cell survival and integration into the patient’s retina [164]. While transplantation of these options is currently being investigated in preclinical animal studies, human clinical trials are primarily testing RPE transplantation for the treatment of RP, SD, and AMD [165]. In NCT06891885, an allogeneic iPSC-derived retinal sheet (DSP-3077), including abundant photoreceptor precursors, is used for patients with RP. Phase 1/2 of the trial will study the safety, tolerability, and clinical responses following DSP-3077 treatment in low and high doses. The long-term therapy effects will be assessed for a total of 60 months. Phase 1 of NCT06789445 focuses on safety and features a dose-escalation design during human iPSC-derived photoreceptor precursor therapy (OpCT-001-101) in patients with primary PR disease. Phase 2 of this trial is designed to gather additional safety data and assess the effects of OpCT-001 on functional vision measures. The initial effects of therapy are assessed for 52 weeks. The observational trial NCT01432847 aims to establish a biospecimen repository for the generation of iPSCs, which will be used to determine molecular mechanisms for potentially blinding eye diseases. It is planned to recruit 465 participants with ocular conditions; their unaffected siblings and relatives will be recruited as healthy volunteers.

MSCs are also used for the cell therapy of retinal diseases [166]. Notably, although some preclinical studies have reported successful use of MSCs differentiated into retinal cells for transplantation, clinical trials typically involve injections of undifferentiated MSCs, which have a therapeutic effect primarily due to their paracrine properties [165]. For example, the two largest studies, the Stem Cell Ophthalmology Treatment Study (SCOTS) and SCOTS2 (NCT01920867 and NCT03011541), were carried out on 300 and 500 patients, respectively. These multicenter studies are investigating the use of autologous BM-MSCs to treat a wide range of conditions, including RP, SD, AMD, optic atrophy, etc. NCT05800301 used subtenon umbilical cord WJ-MSCs alone or in combination with retinal electromagnetic stimulation with the aim of decreasing the natural progression rate of RP. There were no serious adverse events or ophthalmic/systemic side effects for 6 months during the follow-up period; the mean best corrected visual acuity was 70.5 letters prior to WJ-MSC application and 80.6 letters at the sixth month of the observation. In NCT01531348, human BM-MSCs were injected intravitreally into patients with RP. Unfortunately, despite the ‘completed’ status, no results of the trial were provided. According to the results of the NCT01531348 study, where 14 participants had a single dose intravitreal BM-MSC injection, statistically significant improvements in the best corrected visual acuity compared to baseline were established. In the NCT05909488 and NCT03011541 trials on recruiting status, UC-MSC or BM-MSC transplantation was used for RP treatment. In general, clinical trials investigating the safety and efficiency of iPSCs and MSCs for cell therapy for retinal diseases are in the early stages and are mainly aimed at studying adverse reactions and complications, while the long-term effectiveness of these treatments remains to be seen [167].

#### 3.1.2. Neurodevelopmental and Neurodegenerative Diseases

The central nervous system is a complex cellular network consisting of neurons, astrocytes, oligodendrocytes, and microglia. Any disruption in the development or damage to these cells usually leads to serious physiological impairments. In addition to the IRDs described above, hereditary diseases of the nervous system include neurodevelopmental (NDevDs) and neurodegenerative (NDDs) diseases. NDevDs are associated with abnormal development of the nervous system, leading to defects in neural connections or brain architecture, which often entail intellectual disability, seizures, behavioral abnormalities, etc. Diseases of this group include, for example, Rett syndrome (RTT), most often caused by mutations in the *MECP2* gene [168], and autism spectrum disorders (ASD), associated with mutations in more than 800 genes (such as *CHD8*, *PTEN*, *SHANK3*, etc.) [169]. NDDs are characterized by progressive loss of neuronal structure and function, eventually resulting in cellular death, which leads to the gradual deterioration of cognitive, motor, and physiological functions. The most common NDDs include Alzheimer’s disease (AD), Parkinson’s disease (PD) [170], amyotrophic lateral sclerosis (ALS) [171], Huntington’s disease (HD) [172], and others. Although in most cases, these diseases are thought to be hereditary, the exact nature of the underlying genetic abnormalities is known only for 10% of them. For example, hereditary forms of AD are associated with mutations in the *PSEN1*, *PSEN2*, and *APP* genes [173], and the ε4 allele of the *APOE* gene significantly increases the risk of AD [174]. In general, the pathogenetic basis of most NDevDs and NDDs is quite complex, and consequently, these diseases are still not fully understood, making them difficult to treat. This makes the development of clinically relevant in vitro NDD and NDevD models a pressing issue.

2D cultures of MSCs are capable of neuronal differentiation [118,175]. DPSCs are especially responsive to neurogenic induction due to their origin from neural crest cells [176]. Relatively few MSC-based in vitro NDD models have been reported thus far. A recent study reported the creation of an ALS model using AM-MSCs, differentiated into motor neurons and transfected with a plasmid encoding the SOD1 protein with the p.Gly93Ala mutation. Cellular degeneration decreased acetylcholine and glutathione levels, which are characteristic of ALS, have been successfully recapitulated in the model [177]. Several MSC-derived AD models have also been reported. In one study, AM-MSCs were differentiated into cholinergic neurons and then treated with beta-amyloid 1–42 (Aß) to induce neurodegeneration. Screening of both commercially available drugs and two new small-molecule AD drugs was then conducted. The drugs were found to have neuroprotective properties and were shown to restore acetylcholine levels and reduce BACE1 expression [178]. In another study, an AD model was created using cerebral spheroids from menstrual stromal cells (MenSCs) from a patient with the p.Glu280Ala mutation in the *PSEN1* gene. The combination of epigallocatechin-3-gallate and melatonin tested in this model was effective in reducing the accumulation of intracellular amyloid beta protein (AβPP) fragments and attenuating oxidative stress [179]. Thus, in vitro, MSC-based NDD models are a promising platform for drug screening.

iPSCs are capable of 2D and 3D differentiation into neuronal cells and brain organoids (BOs) and are actively used to study the mechanisms of NDevDs and NDDs pathogenesis [180,181]. BOs are particularly attractive in this regard due to their capability of reproducing the main aspects of human brain organogenesis in vivo, as well as the presence of excitatory and inhibitory neurons, astrocytes, oligodendrocytes, and other highly specialized cells in them [182,183,184]. Various differentiation protocols allow the generation of organoids specific to certain brain regions: cortical organoids [185], midbrain organoids [186], thalamic and hypothalamic organoids [187,188], pituitary organoids [189], and many others. These organoids can be studied individually, but can also be combined into assemblies to model complex interactions between different brain regions, such as cell migration and connectivity [190,191]. The emergence of functional neuronal connections is also studied by xenotransplantation of single neurons or BOs derived from human iPSCs into the brains of immunodeficient mice [192]. Similar to ROs, BOs undergo partial necrosis as the organoid enlarges due to insufficient diffusion of oxygen and nutrients due to the lack of both vascularization and microglia. Various methods and examples of organ-on-a-chip and vascularized and immunized organoid production have been recently reviewed [193,194,195,196,197]. Notably, a recent study reported the creation of a complex in vitro multi-organ-on-a-chip system, PEGASO, which incorporated various primary and iPSC-derived cells replicating the functions of the intestine, immune system, liver, blood–brain barrier (BBB), and brain. This platform was used to model the physiological passage of donepezil, an AD drug, and has proven to be an innovative tool for drug screening [198].

Numerous insightful review articles have compiled examples of in vitro modeling of diseases such as AD, PD, ASD, ALS, epilepsy, schizophrenia, etc., based on iPSCs [180,199,200,201,202]. Below, we will provide specific examples of recent iPSC-based modeling of NDevDs and NDDs. For example, a recent study reported the creation of a midbrain and striatum assembloid model from iPSCs genetically modified to overexpress progerin, which induces aging and early cell degeneration. This model demonstrated the development of early neurodegenerative phenotypes associated with PD, which can subsequently be used to study this disease [203]. In another study, a neuromuscular junction (NMJ) model of ALS was created from motor neurons and skeletal myoblasts derived from iPSCs with p.Asp90Ala and p.Gly85Arg mutations in the *SOD1* gene. The critical role of neurons in the development of the NMJ was revealed: the expression of acetylcholine receptors was significantly reduced in them, which ultimately led to impaired muscle contractions [204]. iPSCs are also actively used to test new therapeutic strategies for the treatment of NDevDs and NDDs. For example, neurons obtained from iPSCs of a patient with HD, characterized by 43 and 18 CAG repeats in the *HTT* gene, were then edited using the CRISPR–Cas9 ribonucleoprotein delivery system (RIDE) based on virus-like particles with the VSV-G pseudotype. The editing efficiency reached up to 39% in the indel regions of the HTT locus [205]. In another study, the effect of antisense oligonucleotides was assessed on human iPSC-derived neurons of patients with the *MECP2* duplication syndrome: two-week treatment was sufficient for a high-quality restoration of gene expression and the beginning of improvement in neuronal morphology, but not for its complete restoration [206]. Finally, iPSC-derived neurons from patients with intronic G4C2 repeat expansion in the ALS-associated C9orf72 gene were edited using dual-targeting CRISPR-CasRx, resulting in reduced levels of endogenous sense and antisense repeat RNAs and the prevention of glutamate-induced excitotoxicity [207].

Both MSCs and iPSCs are also considered promising cells for the cell therapy of some NDevDs and NDDs [208], such as AD [209], PD [210], ALS [211], HD [212], and others. Various subpopulations of neurons obtained from iPSCs can be transplanted into damaged brain structures, where they will restore disrupted neural connections. Currently, clinical trials of the safety and efficacy of cell therapy for NDDs using iPSCs are in phases 1–2 [213]. The goal of the now-completed NCT03635294 was to combine genomics, transcriptomics, metabolomics, and morphological analyses of iPSC-derived neural cells from blood samples to improve the diagnosis of an intellectual disability (ID). The main goal of another completed study (NCT02980302) was to obtain an innovative tool (neuronal distinction of iPSCs) that would allow the study of the functional impact of mutations in genes, such as *MYT1L*, probably involved in ID, such as skin cells after cutaneous biopsy being used as the input material. The NCT02720939 study provided a platform to reveal the etiology of the genetic basis and molecular mechanism of ASD. The differences in the cell phenotype of ASD patients and healthy controls were investigated based on peripheral blood mononuclear cell-derived iPSCs.

As for iPSC-based clinical studies aimed at treating NDDs, in the recruiting NCT06821529 study, patients with PD will be treated with autologous iPSC-derived dopamine progenitor cells (iPSC-DAPs) (ICA07). The treatment scheme relies on the injection of 4 million iPSC-DAPs into the putamen on each side of the brain, after which the patient’s status observations will continue for 1 year. The clinical study NCT06778265 is designed to explore the safety and tolerability of a patient’s iPSC-derived mature dopaminergic neurons (UX-DA001), which in the future will be used for the treatment of idiopathic PD. An injectable solution for ALS based on human iPSC-derived motor neuron precursor cells is addressing the pressing need for the treatment of this disorder (NCT06765564). The aim of the NCT06687837 clinical trial is to assess the safety and tolerability of the surgical transplantation of autologous dopaminergic progenitor cells into the brains of participants with PD. The studied characteristics of the cell transplant procedure will be checked through clinical assessments and neuroimaging for 2 years. Allogeneic iPSC-derived dopamine neural progenitors (CT1-DAP001) will be transplanted into the corpus striatum in patients with PD to evaluate the safety and efficacy of treatment (NCT06482268). Another trial (NCT06422208) will assess whether autologous iPSC-derived dopamine neurons from patients with PD are safe when surgically injected into the affected area of the brain. Participants of NCT06344026 will undergo an allogeneic transplantation of iPSC-derived dopamine-producing neurons (ANPD001) into a part of the brain of PD patients where dopamine production is decreased. The effect on PD symptoms, safety, and tolerability, as well as cell survival, will be assessed for 5 years post-transplant. A clinical trial, NCT06145711, also aims to treat PD via autologous transplantation of human iPSC-derived dopaminergic neural precursor cells. In the observational clinical study NCT03322306, hereditary neurological disorder-specific iPSC repositories will be established, registered, and made available to other investigators. The NCT00874783 trial has the common aim of developing iPSCs from patients with neurological diseases for future research.

Similar to the cell therapy of retinal diseases, the value of MSCs for the therapy of NDevDs and NDDs is primarily associated with their neuroprotective and immunomodulatory properties rather than the potential for differentiation into neuronal cells. In this regard, cell-free therapy with MSC extracellular vesicles containing a set of factors secreted by MSCs [214] is of great interest. At the time of writing, only one clinical trial has been registered in which WJ-MSCs are differentiated into neural stem cells (NSCs) and administered to patients with PD (NCT03684122) [215], and the effectiveness of this procedure has not yet been reported. UC-MSCs transplantation will be assessed as therapy against PD progression in the clinical study NCT03550183. The already completed study NCT04821479 demonstrated that the repeated intrathecal administrations of autologous BM-MSCs to ALS patients had no serious adverse events during the entire observation period, and patients had a >25% improvement in the slope of disease progression. The NCT06910384 study assesses the effect of the four doses of autologous BM-MSCs (STR04) on participants with ALS. The primary objective of NCT06858254 is to measure the effects of autologous BM-MSCs transplantation on motor and non-motor functions in patients with PD and Atypical Parkinsonism. Interestingly, the chosen administration route of BM-MSCs is both intranasal and intravenous, delivered in a crossover pattern.

#### 3.1.3. Skin Diseases

Hereditary skin diseases include diseases caused by defects in keratinization, such as ichthyosis [216], various types of keratoderma, and bullous diseases, and in particular, epidermolysis bullosa (EB), which is accompanied by the formation of painful blisters on the skin and mucous membranes [217]. Autosomal recessive congenital ichthyosis (ARCI) is associated with mutations in at least 10 genes, such as *TGM1*, *ALOXE3*, *ALOX12B*, *ABCA12*, and others [218]. Hereditary EB is caused by mutations in at least 16 genes, such as *KRT5*, *KRT14*, *LAMA3*, *COL7A1*, and others [219]. In these diseases, the functions of the main cells of the skin epidermis—keratocytes—are almost always impaired. This results in impaired cell adhesion between keratocytes and disrupts their interactions with fibroblasts and dermal cells.

Currently, primary keratocytes isolated from patient skin biopsy samples, commercially available keratocyte cell lines, and human skin equivalents (HSE) created by culturing keratocytes and fibroblasts in a 3D matrix are most often used for the in vitro modeling of skin diseases [220,221]. The ability to differentiate into keratocyte-like cells (KLCs) has been reported for ASCs, BM-MSCs, UC-MSCs, MenSCs, and other types of MSCs [222]; however, to date, we are not aware of any attempts to utilize these cells to model skin diseases. iPSCs are capable of 2D differentiation into KLCs, as well as the formation of skin organoids (SkOs) with characteristic morphological features, such as hair follicles, sweat glands, and sebaceous glands [223,224,225]. It is assumed that by the 130th day of differentiation, SkOs are able to replicate most of the cellular composition and architecture of fetal skin at 18 weeks of gestation [223], which may be useful for studying the stages of early human skin development. The disadvantage of these organoids is in their structure itself: the growth of hair follicles and exfoliation of the keratinized layer of skin occur inside the organoid, which can potentially be corrected by growing flat skin in organ-on-chip systems. Various methods can be used to vascularize SkOs, including co-culture with endothelial cells, the use of microfluidic devices, and others [137].

There are several examples of in vitro modeling of hereditary skin diseases based on iPSCs. In one of them, basal KLCs were obtained from iPSCs of patients with ARCI associated with mutations in the *TGM1* c.765delT or *PNPLA1* c.736C>T genes and with a form of syndromic ichthyosis trichothiodystrophy (TTD) caused by the c.335G>A mutation in the *ERCC2* gene. After terminal differentiation using high calcium concentrations, ARCI iPSC-derived KLCs demonstrated reduced expression of most of the genes responsible for keratinization, and TTD iPSC-derived KLCs showed reduced expression of *FLG*, *SPRR2B*, and lipoxygenase genes [226]. In another study, EB simplex (EBS) keratocytes derived from patient iPSCs with various mutations in the *KRT5* gene demonstrated decreased proliferation, increased migratory capacity, the presence of cytoplasmic keratin aggregates, and other features of EBS. The potential of this model for testing therapeutic agents was confirmed by the use of 4-phenylbutyric acid, which eliminated some components of the pathological phenotype associated with EBS [227]. A recent study also tested a twin prime editing strategy using the PEmax and recently evolved PE6 prime editors and dual prime editing guide RNAs flanking *COL7A1* exon 5 in iPSCs from patients with recessive dystrophic EB (RDEB) caused by a deletion (c.553C>T) in *COL7A1*. Highly efficient editing of the iPSC genotype was observed, and the edited cells were then shown to be able to differentiate into MSCs and keratocytes, making them a potential cell source for regenerative therapy of RDEB [228].

iPSCs, due to their ability to differentiate into keratocytes and other skin cell types, are currently being actively explored for cell-based therapies for skin diseases. iPSCs represent a virtually unlimited source of cells, whereas primary human keratocytes have limited in vitro proliferation capacity. Several strategies for gene editing of iPSCs for personalized therapies have been demonstrated at the preclinical level. For example, the development of a scalable and cGMP-compliant autologous organotypic cell therapy for RDEB was recently reported, in which *COL7A1* mutation in iPSCs was edited with CRISPR/Cas9 concurrently with their reprogramming into basal keratocytes, dermal fibroblasts, and melanocytes in a single manufacturing step [229]. The use of various multicellular transplants [230], skin-derived precursor cells obtained from iPSCs [231], and extracellular vesicles isolated from iPSCs themselves or epidermal organoids [232,233] are also considered promising therapeutic approaches. MSCs are also used for autologous and allogeneic therapy of skin diseases, mainly because of the regenerative properties of their secretome. Clinical trials of MSC-based approaches have been extensively reviewed elsewhere [234,235]; however, some draw our attention. For example, the safety and efficacy of allogeneic ASCs in a hydrogel sheet (ALLO-ASC-DFU) were assessed in patients with dystrophic EB in the completed NCT02579369 study. The results of another completed NCT03529877 study demonstrated good tolerability, manageable safety, and potential efficacy of intravenous ATP Binding Cassette Transporter B5 (ABCB5)+ MSCs (allo-APZ2-EB) as a readily available therapy for the treatment of RDEB with three adverse events (1 mild lymphadenopathy, two hypersensitivity reactions), which were resolved after withdrawal of treatment.

### 3.2. Mesodermal and Endodermal Derivatives

#### 3.2.1. Hemophilia

Hemophilia is a blood coagulation disorder caused by mutations in genes encoding either the clotting factor VIII (FVIII, hemophilia A) or factor IX (FIX, hemophilia B). Insufficient levels of these proteins in blood plasma result in a failure of the coagulation cascade, which symptomatically manifests as sudden, intense bleeding [236]. This introduces additional hurdles for regenerative therapies: while invasive procedures on patients afflicted with hemophilia are theoretically possible, they require extensive preoperative preparations [237], complicating both the initial cell sourcing and the possible transplantations. The most important aspect of in vitro hemophilia modeling is the activity of the coagulation factors and restoration thereof, which can theoretically be studied in a number of cell types, even the simpler ones, such as HEK293, after performing the appropriate gene editing [238]. Stem cells possess the advantage of being able to be used either directly, like MSCs, or after differentiation and transplantation of the resulting product, like iPSCs, as detailed in the following section. Therefore, meticulously characterizing the activity of coagulation factors after carrying out gene replacement in vitro to prove its efficiency and safety is especially important when working with stem cells.

Owing to the need to minimize invasive procedures on hemophilia-afflicted patients, the least invasive iPSC source material harvesting protocols are preferred, with renal epithelial cells, in particular, being a good fit [239,240]. As far as differentiation is concerned, endothelial cells and hepatocytes are the most common types of derived cells, being responsible for the secretion of FVIII and FIX, respectively, in vivo [241,242]. Grafting the iPSC-derived endothelial cells with the FVIII mutation corrected by CRISPR/Cas9 into the hemophilia mouse models indeed leads to a hemostatic effect, and with robust, optimized differentiation/purification protocols, engrafted cells can survive for months [243]. Some mutations, like the c.947T>C (I316T) in FIX, however, pose additional difficulties due to the lack of a protospacer adjacent motif sequence necessary for the Cas9 nickase to recognize the target sequence. Base editing with engineered Cas9 (SpCas9-NG), which has a requirement of only a single guanine instead of the usual trinucleotide, can successfully target and revert this mutation in patient-derived iPSCs, which showed hemostatic activity after being transplanted into the subrenal capsule of hemophilic mice [244]. Stable expression of coagulation factors can also be achieved via lentiviral transduction, for example, transducing iPSC-derived endothelial cells with a vector encoding the beta-domain-deleted FVIII, which likewise leads to a hemostatic effect after engraftment, even in immunodeficient and neonatal mouse models [245].

Since gene editing techniques are used to correct mutations in patient-derived iPSCs, this presents an opportunity to modify the expressed factors as well, as the half-life of these proteins is relatively low [246]. One of the most commonly used modified factors, F9-Padua, has been shown to be 364% more active than the original FIX protein and can be expressed in iPSC-derived hepatocytes to study the mechanisms of its abnormal activity in vitro and possibly for potential therapeutic use in the future [247]. Modified FVIII has also been reported—insertion of an F309S/E1984V-mutated B domain-deleted-FVIII under the elongation factor-1 alpha (EF-1α) promoter into the AAVS1 locus of the patient-derived iPSCs results in the constitutive expression of an FVIII protein with enhanced activity and stability, both in vitro and after transplantation into mice [248].

The major limitation of 2D iPSC-derived hepatocyte cultures is the incomplete posttranslational processing of FIX in them, likely owing to the immature phenotype of such cultures. FIX produced by 2D iPSC-derived hepatocyte cultures lacks γ-carboxylation of the GLA-rich domain and has greatly reduced activity as a result. In contrast, mature hepatocytes, marked by the presence of *CYP3A4*/*2B6*, *SRB1*, *CK8*, *CX32*, *MDR3*, and *BSEP*, generated as constituent parts of organoid cultures, are able to synthesize this protein correctly and thus serve as a superior model of FIX production and activity [249].

Compared to iPSCs, gene-edited MSCs are capable of engraftment without additional differentiation, requiring only the appropriate knock-ins or mutation editing if taken from a hemophilic patient. Using MSCs as gene carriers, as opposed to direct viral vector injection, can help avoid viral vector exposure and, for example, may help reduce organ-specific toxicity and the potential risk of the immune response associated with high AAV vector doses, especially when non-viral promoters like EF-1α are used [250,251,252]. Coagulation factor secretion can also be achieved without genetic engineering by the in vitro conditioning of MSCs towards endothelial lineages. Commercial ASCs, which normally release FVIII only in minor quantities, can be induced to upregulate their secretion after treatment with a conditioning media with inductive factors (VEGF, hFGF-b, EGF, and IGF-1), with longer treatment resulting in better plasma coagulation, as measured by an in vitro activated partial thromboplastin time test [253]. Additionally, human amniotic fluid MSCs have been shown to integrate successfully into the liver of neonatal mice after in utero transplantation, even fusing with mouse cells and retaining their native capability to secrete FVIII. This makes in utero MSC therapy a promising strategy for treating hemophilia in newborns, in which other methods, such as gene therapy through non-integrating vectors, are not as effective due to the “dilution” of the expression with each cell division [254].

However, obtaining human MSCs for autologous treatment of hemophilia has an unfortunate complication: due to most MSC isolation procedures being relatively invasive, a patient’s own MSCs may not be realistically obtainable. A potential solution to this problem would be to differentiate MSCs from iPSCs—it has been shown that such iPSC-derived MSCs with an edited FVIII mutation are just as capable of achieving long-term engraftment and producing functional FVIII, at least in mice, and may eventually become a viable alternative for humans, as well [255]. However, while no iPSC-based clinical trials for the treatment of hemophilia have been registered thus far, the NCT02108132 trial is assessing the effects of BM-MSCs from healthy donors induced to adopt the hepatocyte phenotype and injected into hemophilia patients through the portal vein. The goal of another MSC-based study (NCT05187936) is to show preclinical interest in MSC therapy in hemophilic arthropathy, which is one of the most frequent complications in hemophiliacs.

#### 3.2.2. Marfan Syndrome

Marfan syndrome (MFS) is a multisystem disorder caused by mutations in the *FBN1* gene and, to a lesser extent, in *TGFBR2*. As fibrillin-1 stabilizes latent transforming growth factor β-binding proteins (LTBPs) in the ECM, its deficiency also causes abnormal levels of TGF-β activity. MFS predominantly affects connective tissue in the cardiovascular system, eyes, and skeleton, with the leading cause of mortality being the dilatation and subsequent dissection or rupture of the aorta [256].

iPSC-derived in vitro models are well-established in MFS modeling, where cardiomyocytes and vascular smooth muscle cells (VSMC) are typically used, with at least 52 iPSC lines harboring distinct MFS-causing mutations being described in published studies and uploaded to online stem cell registries, as reviewed in [257]. Recent investigations into the molecular mechanisms of the MFS with the iPSC-derived cells have helped clarify the role of multiple signal transduction pathways in the pathogenesis of this disease [258]. A great advantage of iPSCs in this regard is their ability to differentiate into VSMCs of different embryonic origins, allowing the direct in vitro investigation of the differing impact the MFS-causing mutations may have on them. While it was previously known that upregulated integrin αv signaling was implicated in the development of aortic root aneurysms, its role as a potential disease marker was not confirmed until recently. Integrin αv was shown to be upregulated along with its downstream targets, FAK and Akt, in iPSC-derived MFS VSMCs differentiated into both the cells of the second heart field (SHF-VSMCs), residing in the aortic root, and the cells of the neural crest origin (NC-VSMCs), residing within the ascending aorta. FAK and Akt upregulation, in turn, activated the mTOR pathway, as confirmed by immunoblotting and quantitative ELISA. Administration of the integrin αv receptor blocker to a mouse model of MFS was able to attenuate the progression of the aortic aneurysm, confirming the role this pathway plays in the etiology of MFS [259]. Another novel molecule that was found to play a role in aortic aneurysm development is GSK3β, which was repeatedly identified in a screening of small molecules capable of reducing the matrix-metalloprotease activity of VSMCs. Inhibition of GSK3β was shown to reduce apoptosis and proteolysis in MFS VSMC cultures derived from multiple different patients and additionally corrects the abnormal fibrillin-1 deposition associated with MFS, potentially identifying it as one of the key components of the disease phenotype [260].

The modeling of local heterogeneity in the aorta can be further improved by the utilization of microfluidic chip devices, which allow the simulation of hydrodynamic tensile forces, creating an environment closely resembling that of the original vessel. For example, it was shown that VSMCs derived from the paraxial mesoderm are more sensitive to tensional stress than those of lateral mesoderm and neural crest origin, likely owing to differences in the PI3K-Akt signaling pathway [261]. 3D cultures, such as the MFS cardiospheres, composed of iPSC-derived cardiomyocytes and fibroblasts, have also been reported, and allow more representative modeling of cell-to-cell and cell-to-ECM interactions [262].

iPSC-derived VSMC cultures can also serve as a model for investigating the aberrant biomechanical interactions of the MFS-affected cells with the extracellular matrix. MFS SMCs have been shown to be affected by the substrate stiffness to a greater degree than their wild-type counterparts—nuclear localization of YAP, indicative of the active YAP/TAZ signaling pathway, and the formation of actin stress fibers, have been shown to occur on substrates with a stiffness as low as 12 kPa, compared to the 50 kPa required to induce the same phenotype in wild-type cells [263]. Furthermore, the stiffness of the VSMCs themselves has also been shown to vary in a region-specific manner: healthy and MFS iPSCs differentiated into region-specific VSMCs show a different response when probed for stiffness using atomic force microscopy. Healthy VSMCs from the ascending aorta tend to be stiffer than the ones from the descending aorta, whereas the opposite is true for the MFS VSMCs. These intrinsic aberrant mechanical properties likely make them more vulnerable to hemodynamic stress, potentially providing an explanation for the predominantly ascending localization of MFS-related aortic aneurysms [264]. Another potential reason for this localization is the region-specific deficiency in Mannose Receptor 2 (MRC2) expression, an important component of collagen homeostasis. iPSC-derived SHF-VSMCs show a significantly lower level of MRC2 expression compared to the NC-VSMCs, leading to the overexpression of COL1a1 and the consequent reduction in collagen turnover. These results have been confirmed in mouse models of MFS, where an MRC2 deficiency was shown to result in the enlargement of the aortic root, potentially compromising the integrity of the aortic wall [265]. The upregulation of genes responsible for elastin fiber formation and cell adhesion in response to estrogen has also been observed in iPSC-derived VSMCs, providing a potential clue to the molecular basis behind the sex-specific phenotypes seen in some cardiovascular disorders [266]. Finally, MFS iPSCs with the deletion of the entire *FBN1* gene have been used to model the behavior of embryoid bodies (EB) in microgravity conditions, simulated by the Desktop Random Positioning Machine, which nullifies the average gravity vector through continuous random change in orientation. After 120 h in simulated microgravity, MFS EBs showed a more disordered, irregular morphology as well as a reduced adaptive and migratory capacity compared to the wild-type EB, presumably due to the structural deficiency caused by the lack of fibrillin-1 [267]. Generation of MFS and Fontan cardiovascular models using patient-specific iPSCs became the main goal of the observational NCT02815072 study, which might give further clues into the manifestation of structural heart disease.

Surprisingly, despite the evidence that MSCs have an altered phenotype in MFS, we are not aware of any papers that have attempted to use primary MSCs as an in vitro model of MFS or even therapeutically. MFS seems to primarily affect MSCs in the aortic wall, which have been shown to have a downregulated expression of LTBP1, a key component of the latent TGF-β complex, contributing to the general dysfunction of this pathway [268]. Local loss of TGF-β signaling has also been associated with fibrillin-1 deficiency, at least in the perichondrium [269]. Furthermore, fibrillin microfiber formation in MFS-affected and mutation-corrected MSCs has been compared in vitro; however, both types of MSCs in this particular case have been differentiated from patient-derived iPSCs [270]. Taken together, these results suggest that despite it being relatively underappreciated, there is some potential for in vitro modeling of MFS using MSCs.

#### 3.2.3. Cystic Fibrosis

Cystic fibrosis (CF) is an autosomal recessive monogenic disorder arising due to mutations in the cystic fibrosis transmembrane conductance regulator (*CFTR*) gene, which encodes a cAMP-dependent chloride ion channel. Various mutations in *CFTR* result in abnormally viscous secretions in the organ systems containing epithelia, most notably the lungs, pancreas, and parts of the digestive system, leading to obstruction, inflammation, and tissue damage in affected organs [271], as well as increasing the risk of respiratory infections [272,273].

CF is typically studied in vitro using the cells from nasal, bronchial, and rectal epithelia [274]. Most of these models tend to utilize primary cells despite their low yields and the relative lack of potential for scalability. While this issue could potentially be circumvented through the use of iPSC-derived cells, surprisingly few studies on the etiology of CF have utilized iPSCs thus far, despite it being the most widespread monogenic respiratory disease, resulting in a marked heterogeneity in published data [275].

iPSC-derived progenitor lung cells grown in 2D culture have been shown to adopt transcriptional characteristics intermediate between the iPSCs and differentiated mature bronchial epithelium. Notably, these cells demonstrate upregulated expression of genes associated with early club cells and downregulation of genes associated with mature cells (such as the *SCGB1A1* and *MUC5AC*), suggesting a relatively immature phenotype. However, the elevated *CFTR* expression still makes them a suitable model for analysis of the channel function in response to different mutations and treatment strategies, for example, via the fluorescence-based assay of membrane potential changes (FLIPR) [276]. The activity of CFTR in iPSC-derived airway epithelial spheroids can also be assessed through the forskolin-induced swelling (FIS) assay, which measures the kinetics of spheroid swelling after the CFTR activation through adenyl cyclase stimulation by forskolin, making this model particularly relevant for physiological studies [277]. Validation of an in vitro iPSC-based model of CF created using differentiated bronchial epithelium from CF patients was carried out in an observational clinical study, NCT03754088.

Furthermore, the generation of specific rare cell types present in the lung epithelium is also possible, making it easier to unravel their individual contributions to the CF phenotype. Pulmonary ionocytes are one such cell type that is characterized by their elevated expression of CFTR and the presence of FOXI1 and ASCL3 transcription factors. These cells have been shown to be generated in a mixed airway epithelial culture after pulmonary differentiation and two rounds of sequential sorting of airway basal-like cells for NGFR at a 1% frequency, consistent with the frequency of their primary counterparts in human large airways [278].

Another CF model system is the intestinal epithelium. While intestinal organoids used in CF research can be derived from biopsies and have been shown to be effective in FIS assays as well [279], the invasiveness of this process makes iPSC-derived intestinal organoids an attractive alternative while still allowing the patient-specific genotype to be maintained. Furthermore, by removing the supporting extracellular matrix, these organoids can be split open, transforming them into a 2D culture suitable for electrophysiological assays of CFTR modulator activity [276].

Since CF affects multiple tissues, some studies have attempted to compare the results obtained in the iPSC-derived airway and intestinal epithelia in order to predict the organ-specific drug response. For example, the pharmacological rescue of the F508del *CFTR* mutation using the Trikafta modulator, as measured by the apical chloride conductance assay, has been shown to be more effective in the colon tissue compared to the lung tissue [280]. Due to the systemic nature of CF and the inability of the rodent models to recapitulate the most detrimental clinical aspects of the disease, particularly in the lungs, microfluidic organ-on-chip devices can serve as a powerful tool to analyze the more complex interactions of the diseased tissues [281].

In contrast, comparatively little research on CF has thus far utilized MSCs, with recent clinical research primarily focusing on alleviating inflammation and bacterial infections in lungs [282], and has so far been met with only limited success, perhaps partially owing to the incomplete understanding of the exact effects MSCs have on the lung microenvironment [283]. Other promising results include the finding that amniotic MSCs exhibit pronounced regenerative properties when cultured together with bronchial epithelial cells in an in vitro wound-healing assay, thought to be mediated by the downregulation of CX43 [284], as well as the report of MSCs being successfully used as a vehicle for the exosome delivery of CFTR zinc finger protein fusion, capable of reactivating the CFTR expression in human bronchial epithelial cells, at least in vitro. Another completed study, NCT02866721, tested the safety of allogeneic human MSC therapy at 1 × 10^6^, 3 × 10^6^, or 5 × 10^6^ cells/kg of body weight delivered to adult CF patients. Unfortunately, no patients were recruited for the NCT03058068 study, aimed at assessing the effects of MSC delivery in order to improve the symptoms of CF, including lung function, the rate of pulmonary exacerbation, systemic and local inflammation, and symptom-related quality of life, and thus, it had to be withdrawn.

Curiously, despite MSCs being shown to be capable of differentiating into epithelial-like cells, both after co-culture with lung airway epithelium cells [285,286] and conditioning with the media from intestinal epithelium cells [287] and, in both cases, acquiring parts of their respective phenotype; to our knowledge, no attempts have been made in recent years to exploit this capability for the in vitro modeling of CF.

#### 3.2.4. Muscular Dystrophies

Muscular dystrophies (MDs) are a group of inherited muscle diseases that share the common clinical feature of the progressive weakening of muscles, as well as their characteristic dystrophic appearance on biopsies. The pathogenetic basis of these diseases varies to a great extent, from mutations in the extracellular matrix proteins, such as the three collagen 6 alpha chains associated with the Ulrich congenital muscular dystrophy (UCMD) and limb-girdle dystrophy, to mutations in the sarcolemma-associated proteins, which stabilize muscle fibers against the contractile force. In particular, mutations in dystrophin, one such protein, result in Duchenne muscular dystrophy (DMD), the most common form of muscular dystrophy [288].

No universal therapeutic strategy exists for these diseases as of this writing, making the development of suitable models a particularly pressing issue. The most suitable cell types, myoblasts and cardiomyocytes, are difficult to obtain and have limited proliferative capacity in culture, further hampered by the MD genetic background. This can be overcome by the use of their iPSC-derived counterparts. Myogenic differentiation in iPSCs can be induced through the activation of Wnt signaling and/or inhibition of bone morphogenic protein (BMP) activity with small molecules or with the overexpression of muscle-specific transcription factors, namely PAX3, PAX7, and MyoD. Both approaches can be used in conjunction. Cardiomyocytes are significantly more difficult to derive and first require the generation of cardiac mesodermal progenitors through induction with nodal, BMP, and fibroblast growth factors (FGFs) [289]. Furthermore, new approaches towards efficient and scalable iPSC differentiation into isogenic muscle disease models are constantly being developed, both for the generation of 2D cultures and the 3D tissue-engineered cultures, suitable for tasks such as the measurement of the contractile forces [290,291].

No successful clinical translations of iPSC-based MD therapeutic approaches have been reported thus far, with most studies being confined to a purely academic environment [292]. The only clinical trial (NCT02413450) using iPSCs for the treatment of MDs is observational and will collect blood or skin biopsies from patients and healthy donors with the purpose of generating cell and tissue models of heritable forms of heart disease, such as cardiomyopathies, channelopathies, and neuromuscular diseases.

iPSC-derived cardiomyocytes were shown to exhibit telomere shortening, which is associated with multiple morphological and physiological defects and does not occur in the CRISPR-corrected isogenic controls. The overexpression of the *TRF2* gene, encoding a key shelterin protein, was able to prevent telomere attrition and improve the viability of these cells [293]. Proof-of-principle gene editing experiments are also common. Since many DMD mutations occur after exon 44, some studies attempt to “trim” the protein in order to remove the disease-causing exons altogether without impairing its function. Gene-edited DMD iPSCs with a DMD knock-in that connected exon 44 directly with exons 58–70 were found to differentiate into the dystrophin-expressing myotubes both in vitro and after transplantation into mdx (DMD model) mice [294], and similar results have been achieved by deleting exons 48–54, which resulted in the functional beating iPSC-derived cardiomyocytes [295].

Attempts have been made to use iPSC-derived cells directly for transplantation. Human iPSC-derived muscle stem cells, transplanted into the dystrophic mouse diaphragm, managed to successfully produce dystrophin fibers after engraftment, partially restoring the diaphragm function. Despite the overall limited engraftment efficiency, promising results have been achieved by injecting the cells together with the hyaluronic acid–gelatin polymer mixture at a ratio of 8:2, which significantly improved the retention of cells in the rapidly moving diaphragm [296]. The fetal phenotype typical of the iPSC-derived cells does not hinder the efficiency of transplantation; in fact, it has been shown by transcriptomic analysis that in vitro-derived iPAX7 myogenic progenitors undergo maturation upon being transplanted into mice, notably showing no significant difference in maturation between the DMD and healthy mice [297]. iPSC transplantation for DMD cell replacement therapy also holds potential for the amelioration of hypoxia-induced muscle damage. In an in vitro transwell co-culture model, iPSCs were shown to reduce ROS levels and decrease hypoxia-induced LDH release in C2C12 myoblasts, enhancing their viability under hypoxic conditions [298].

The retention of patient-specific genotypes in iPSCs has also allowed the study of the molecular mechanisms of rare MDs. Emery–Dreifuss muscular dystrophy type 1 (EDMD1) arises due to mutations in the *EMD* gene, which encodes emerin, a nuclear protein implicated in nuclear stability, genome organization, and regulation of cell cycle and gene expression. The exact details of emerin function and regulation are species-dependent, limiting the utility of animal models, while human muscle biopsies have only been able to provide limited amounts of data due to their rarity. The establishment of an iPSC-derived model promises to overcome said issues in the future. iPSC-derived EDMD1 myoblasts reflect the delayed differentiation timing associated with the disease phenotype and display altered activities of Akt, Wnt/β-catenin, MAPK/Erk, IGF-1, TGF-β, and Notch signaling pathways, the consequences of which can now be elucidated in vitro [299]. Rare variations in otherwise relatively well-characterized dystrophies can be studied in greater detail in this way as well. One notable example of this approach is the generation of an iPSC-derived cardiomyocyte model from a young female Becker muscular dystrophy carrier with a rare in-frame heterozygous deletion in exons 45–48 (Δ45–48) of the *DMD* gene. The inability of conventional genome editing to correct the large exon deletions and the localization of the affected gene to the single active X chromosome make the generation of isogenic iPSCs from female patients with DMD mutations a particularly daunting task. The authors overcame this issue by generating iPSCs from peripheral blood lymphocytes, which exhibit a random X-inactivation status [300].

The mechanobiological aspect of the MDs can be relatively easily assessed in vitro due to the ease of access to the substrate. Contractile decline due to muscle fatigue can be modeled with electrical field stimulation (EFS) of the skeletal muscle cell cultures. Short-term EFS has been shown to promote myogenic differentiation and maturation of DMD iPSC-myotubes by mimicking exercise through continuous contraction; however, long-term stimulation leads to a decline in their contractile performance, allowing researchers to recapitulate this particular aspect of the disease in further translational studies [301]. YAP activity has also been shown to be impaired in DMD iPSC-derived cardiomyocytes, estimated by measuring the response to the changing substrate stiffness, which, together with the disruption of the actin stress fibers, may contribute to the disease phenotype [302]. Substrate micropatterning can also be used to improve the contractile and structural maturity of the iPSC-derived cardiomyocytes, which otherwise tend to exhibit a relatively fatal phenotype. Compared to cells cultured in monolayers, iPSC-cardiomyocytes cultured as single cells on patterned substrates exhibit a progressive contractile response towards the increasing concentrations of the contraction-simulating drug isoproterenol, unlike the all-or-nothing response seen in the monolayer. This allows for the measurement of the cell-intrinsic contractile properties, making this model a suitable platform for the study of the effects that different drugs exhibit on the cardiomyocyte contraction [303].

Another biophysical aspect of MDs that is more convenient to study in vitro is the electrophysiological changes in the affected contractile cells. The fetal phenotype of iPSC-derived cardiomyocytes grown in a 2D culture normally precludes such studies, as their underdeveloped sarcoplasmic reticulum cannot facilitate calcium release in sufficient quantities [304]. Once again, substrate micropatterning can be used to circumvent this issue: iPSC-derived DMD cardiomyocytes grown on micropatterned PDMS adopt the typical cylindrical, binucleated morphology with well-developed sarcomeres, which facilitates electrophysiological maturation. This has been applied, for example, to study one of the potential mechanisms for arrhythmia observed in DMD patients, which has been suggested to be the disruption of interactions between the NaV1.5 and Kir2.1 ion channels by the truncated mutant form of dystrophin. The absence of dystrophin has been shown to result in the reduced traffic of both proteins to the cellular membrane, and was reversible through the overexpression of α1-syntrophin, restoring the normal electrical function of the cells [305].

The effect the MD mutations have on the metabolism of individual cell populations can also be assessed in iPSC-derived cells. The loss of dystrophin in DMD iPSCs was shown to result in a lower expression of respiratory complex proteins and a change in the mitochondrial morphology—the mitochondrial network in DMD iPSCs appeared significantly less branched and elongated compared to control cells, possibly because of the altered actin dynamics. This was accompanied by a change from oxidative phosphorylation to glycolysis and an increased tendency towards myofibroblast differentiation, providing a new link between the increased risk of cardiac fibrosis observed in DMD patients and the molecular basis of the disease [306]. Impaired, depolarized mitochondria have similarly been observed in iPSC-derived cardiomyocytes, which were found to have reduced lifespans in culture due to the damage caused by the elevated levels of reactive oxygen species (ROS). The *NOX4* gene, encoding the NADPH oxidase 4, an enzyme capable of generating ROS as a side effect, was found to be overexpressed in these cells and is proposed to also contribute towards this phenotype. The addition of idebenone to the cardiomyocyte culture seemed to reduce the NADPH-dependent ROS production by NOX4 and even resulted in the repolarization of the mitochondrial membranes [307].

In the context of MDs, MSCs are most commonly used for cell therapy, competing with a number of other diverse cell sources reviewed in [308]. MSCs are thought to be able to transdifferentiate into myogenic lineages and to promote regeneration. WJ-MSCs are considered to be especially attractive as a cell source for MD MSC therapy due to their reduced immunogenicity and superior secretory properties. The multisystem mechanism of WJ-MSC therapeutic action is thought to partially depend on the restoration of the expression of miR-499-5p, a microRNA that normally targets key fibrotic factors TGFβR1 and TGFβR3 [309]. Recent clinical data show that MD treatment in patients with naive WJ-MSCs results in a significant improvement in muscle strength in 54.5% of the patients (12 out of 22 surveyed) and can even ameliorate the symptoms completely in 27.3% of cases (6 out of 22), with no significant side effects being reported [310], with the phase I clinical trials of the allogeneic early passage WJ-MSC (EN001) also confirming their safety during the 12-week period post-injection [311]. Other studies suggest that MSCs derived from iPSCs, specifically those induced in a xeno-free manner (XF-iMSCs), might present an even better alternative, at least for the treatment of UCMD. These XF-iMSCs have been shown to promote the differentiation of MuSCs derived from *Col6a1*-KO mice in an in vitro co-culture, primarily through IGF2 secretion, while their use in an in vivo mouse model demonstrated reduced fibrosis levels at the site of implantation and resulted in an efficient regeneration of muscle fibers. Muscle fibers in the XF-iMSC-transplanted group tended to exhibit a higher cross-sectional area and had a larger percentage of mature regenerated myofibers with two or more nuclei when compared to the results obtained in the ASC and BM-MSC-transplanted groups [312,313]. In the NCT02285673, NCT02235844, and NCT01610440 studies, the efficacy of human UC-MSCs in DMD patients will be assessed. The NCT06328725 trial will check the efficacy and safety of allogeneic early-passage WJ-MSCs (EN001) in DMD patients.

Furthermore, many groups have also attempted to modify MSCs in order to increase the effectiveness of the treatment, most commonly to increase their retention in the tissue by attempting to enhance their aggregation, proliferation, or differentiation efficiency. However, most studies only modified MSCs in a single way, with little overlap between them, making a systematic comparison of modified MSCs and their therapeutic potential limited. Furthermore, very few studies have attempted to test modified MSCs in humans [314]. For example, MSCs modified by AAV1 transduction to continuously produce IL-10 have been demonstrated to have a higher engraftment potential and improved inflammation attenuation both in mice and in beagle dog models of DMD [315]; however, to our knowledge, no attempts have been made to further translate this finding into the clinical practice of treating at least the MDs.

Myoblasts and MSCs are known to fuse upon myotube formation, which can be modeled in vitro. This has utility both for in vitro disease modeling, where the impact of this phenomenon (or lack thereof) on disease progression can be conveniently assessed in an MSC-myoblast co-culture [316], and for the development of potential therapeutic strategies, where fused ex vivo dystrophin-expressing chimeric (DEC) cells are seen as a potential alternative for the conventional MSC treatment, combining the dystrophin-secreting property of myoblasts and the proliferative and secretory capacity of MSCs, while being poised to be effective regardless of the disease-causing mutation [317]. Other beneficial effects, such as the eventual acquisition of healthy mitochondria by DEC cells via chimeric mitochondrial fusion, have also been observed [318]. The first clinical trial of DEC cells, DT-DEC01, is currently underway and has thus far been reported as both safe and effective, at least in the 12-month follow-up period [319].

#### 3.2.5. Bone and Cartilage Disorders

Bone and cartilage defects are a heterogeneous group of disorders. Some, like osteogenesis imperfecta (OI), are primarily caused by mutations in structural proteins of the extracellular matrix, type I collagen in this case [320], whereas others are caused by mutations in components of the major signaling pathways, such as in the *FGFR3*, which causes achondroplasia (ACH) [321].

A major advantage of iPSC-based bone modeling lies in the fact that bone has multiple embryonic sources, which give rise to osseous tissues with different properties—the axial skeleton is produced by the paraxial mesoderm, limbs are derived from the lateral plate mesoderm, and facial bones are a product of the neural crest. By guiding the iPSC along these developmental trails, representative models of each bone lineage can be established [322].

iPSCs can be used to study the effect of specific mutations in bone and cartilage tissues [323,324]. iPSC-derived chondrocytes, for example, have demonstrated varying levels of unfolded protein response (UPR) and endoplasmic reticulum (ER) stress in response to different mutations in the *MATN3* and *COL10A1* genes, likely due to associated disruptions in protein folding. MATN3 R209P mutants were observed to suffer from the most severe enlargement of the ER, while the MATN3 T120M mutants exhibited the most drastic upregulation of chaperone and downstream UPR gene expression [325]. Gene correction of iPSC-derived osteoblasts has also been demonstrated. CRISPR/Cas9 editing of a deletion (c.2423delT) in exon 36 of the *COL1A1* gene in OI-iPSCs was able to ameliorate their otherwise impaired capability for osteogenic differentiation and shows promise as a possible treatment for OI [326]. Similar approaches have been demonstrated for the iPSC-derived chondrocytes as well by restoring the chondrogenic differentiation capacity of ACH iPSCs after the correction of the mutation in the *FGFR3* [327]. Furthermore, culturing patient-derived iPSCs under shaking conditions in an osteogenic induction medium with the addition of retinoic acid results in the formation of 3D bone constructs, which are particularly advantageous for the in-depth studies of mineralization impairment, found, for example, in patients with hypophosphatasia [328]. Promising results have also been shown in the field of cartilage organoid generation, reviewed in [329]. Even more complex bone models can be manufactured using the organ-on-chip approaches, as reviewed in [330].

In addition to being commonly used in cell therapy [331,332], MSCs are also sometimes used to study bone and cartilage diseases in vitro. The osteogenic differentiation of MSCs in vitro is one of their key properties; however, this does not necessarily mean that the ossification mechanism is the same as in an in vivo environment, which is important to remember in the context of using MSC cultures as models for bone diseases, as currently, no cell culture environment can directly replicate all factors involved in osteogenesis, as reviewed in [333]. MSCs derived from OI patients were found to be prematurely committed to osteogenic differentiation. However, they also rarely progressed past the early osteoblast stages. Galunisertib, which blocks the TGF-β pathway by inhibiting the TGF-β receptor I, was able to enhance the maturation process, allowing the cells to reach mature phenotypes [334]. Some studies also utilize patient iPSC-derived MSCs when direct acquisition of MSCs is not feasible. For example, the pathogenetic effects of patient-specific glycine substitutions in *COL1A1* and *COL1A2* have been studied in this way. OI iPSC-derived MSCs showed a significant upregulation of UPR gene expression, which was able to be ameliorated with the administration of the 4-PBA drug to the cell culture, resulting in reduced apoptosis levels, suggesting that attenuating the EPR stress can serve as one of the potential treatment strategies for OI [335]. iPSC-MSCs also bring the ability of iPSCs to model the specific bone lineage into the context of MSC osteogenic differentiation. MSCs differentiated from iPSC-derived intermediate neural crest cells exhibit the same relative expression of MSC markers CD73, CD105, and CD90 compared to the primary MSCs and still exhibit reduced osteogenic differentiation capacity with the OI genotype [336], potentially allowing the impact of particular bone lineage on the differentiation process to be studied in the future.

As of writing this, no clinical trials, either iPSC- or MSC-based, have been registered for these diseases.

#### 3.2.6. Metabolic Disorders

iPSCs are particularly advantageous for modeling the inborn errors in metabolism, as in most cases, the underlying physiological effects do not seem to affect their reprogramming, maintenance, and differentiation processes, likely because these stages recapitulate early embryonic development, and most metabolic diseases are not developmentally lethal [337].

Phenylketonuria (PKU) is the most common disorder caused by inborn errors in amino acid metabolism. The pathogenetic basis of the disease is the mutation in the *PAH* gene, encoding the phenylalanine hydroxylase enzyme, which converts phenylalanine into tyrosine. Loss of PAH activity results in increased concentrations of phenylalanine in the blood and brain and is especially dangerous for the latter—increased levels of phenylalanine in the brain were shown to result in developmental problems and mental impairment [338].

While PKU, being a relatively rare disease, is comparatively uncommonly modeled with iPSCs, established mutation-carrying cell lines do exist [339,340], as well as the precedent for their use. As is typical for metabolic disease, PKU affects multiple tissues and requires multiple cells and even tissue types to capture the systemic complexity, making iPSC-derived models a suitable tool for this purpose, as reviewed in [341].

Interestingly enough, due to the primary effect of the PKU-causing mutation being the aberrant accumulation of phenylalanine in tissue, one does not necessarily have to alter the genome of cells to include it—simply adding the compound to the culture medium was shown to be enough in order to simulate the neurotoxic effects of PKU in cerebral organoids, which was shown to result in impaired organoid growth and partial demyelination. While this approach produces shorter exposures to higher concentrations of phenylalanine compared to those found in vivo, it is still capable of replicating major hallmarks of the phenotype [342]. Another remarkable recent paper demonstrates the use of a bicompartmental enteric organ-on-chip as a model for assessing the safety and efficiency of PKU treatment with synthetic bacteria designed to express the phenylalanine-degrading enzyme PAL. No significant negative effects have been observed after administering a single dose of synthetic bacteria to the gut compartment, and the levels of phenylalanine in the blood compartment were shown to have decreased after that, providing a promising potential treatment strategy [343].

Comparatively, few attempts have been made to model PKU using MSCs, with all of the work in recent years being done by Drs. Harry Blair and Steven Dobrowolski and their collaborators, as part of a series of investigations on the altered bone growth in Pah^enu2^ mice. Specifically, it has been shown that hyperphenylalaninemia causes reduced bone growth through a somewhat unclear mechanism, which is seemingly partly dependent on the reduced osteogenic differentiation of bone MSCs [344]. ROS formation was found to be a major factor. In vitro quantification of superoxide in bone-derived PKU MSC cultures has shown a significant increase in its representation compared to control cells, while high-resolution oximetry showed aberrant oxygen consumption, suggesting oxidative stress and the inability to support osteogenic differentiation [345]. Another piece of evidence for the energy deficit being the major cause behind the bone loss is the improvement of bone density via the administration of glutamine energy substrate, which was found to improve the efficiency of osteogenic differentiation both in vivo and in vitro in MSC cultures, increasing the oxygen consumption of mitochondria in the process [346].

Similar to the bone and cartilage disorders field, the area of PKU is yet to see registered clinical trials taking advantage of the unique features of iPSCs and MSCs.

## 4. Discussion: Future Perspectives of iPSC- and MSC-Derived Disease Model Development

From the analyzed studies, it is evident that both iPSCs and MSCs, despite their many shared properties, are used in rather distinct capacities, both when it comes to in vitro disease modeling and clinical applications.

The use of MSCs as a platform for disease modeling is considerably less frequent compared to their iPSC counterparts. It seems that MSCs are predominantly used in in vitro studies when they are themselves either a key component of the pathogenesis or are later used therapeutically. Their efficacy needs to be tested beforehand. In contrast, very few studies exploit their in vitro capacity for multilineage differentiation in order to create models of diseased (or even healthy) tissue, as is commonly done with iPSCs, even when this approach might be theoretically feasible. It is plausible to speculate that the reduced differentiation capacity of MSCs in comparison to iPSCs is the primary cause of this discrepancy. Even though some diseases can theoretically be modeled with MSCs, the comparatively more relevant iPSC models are seen as preferable, notwithstanding their higher costs and more difficult production. For many years, MSCs have been primarily used for their regenerative potential, immunomodulatory and anti-inflammatory properties, and also, for example, as cell fate-defining feeders for T lymphocytes and HSCs [347,348]. Their primary use in regenerative medicine is reflected by the virtual absence of MSC-based clinical trials testing their application for in vitro disease modeling. In contrast, iPSCs were developed primarily for this purpose. Nevertheless, MSCs are easily accessible and significantly faster and cheaper to obtain and sustain, transform into several lineages, have low tumorigenicity, and are easier to transfect and transduce compared to iPSCs, all of which makes them an attractive platform for in vitro disease modeling, reflecting their inclusion in this review. The final choice of the cell platform is individual and depends on the specific application, cell accessibility, and project funding.

The number of clinical trials focused on cell therapy is steadily increasing (Table 1).

**Figure 3:**
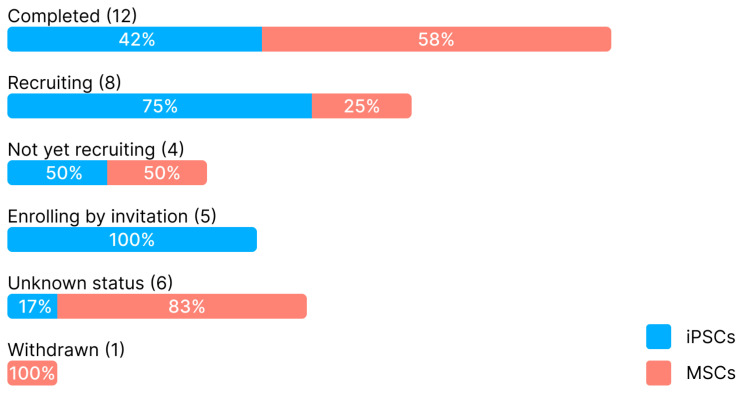
Comparison of iPSC- and MSC-based clinical trials, mentioned in this review.

The distribution of the analyzed trials shows that there is an approximately equal number of MSC- and iPSC-based studies (Figure 3), yet in recent years, greater interest has accompanied the field of iPSC research. Almost 80% of the analyzed studies are interventional, which indicates the practical application of the cell therapy products, with therapies directed toward the treatment of NDDs attracting the most interest. Unfortunately, the outcomes of the studies are rarely publicized, making it extremely difficult to assess the success (or the failure) of the individual therapeutic approaches. The only indirect indication of these is the completed or withdrawn status of the trial. As such, 50% of trials have been completed (with an unknown outcome).

Even the seemingly similar aspects of both cell types are largely distinct upon closer inspection. For example, while both iPSCs and MSCs are considered to be largely heterogeneous, the degree of this heterogeneity is rarely compared directly, despite it having important implications for their use in certain applications requiring high reproducibility, such as drug screening. It would seem that iPSCs, as outlined at the beginning of this review, have more potential sources of heterogeneity—in addition to carrying over the individual genetic differences of the donor, said differences might be inconsistently represented down to the level of individual cells within the iPSC culture due to the incomplete reprogramming efficiency. Furthermore, these differences can be further exaggerated with the arising genomic instability characteristic of iPSC cultures, which is already evident even during the early passages [349,350]. Crucially, most of these differences occur later as a result of the reprogramming process. In contrast, while MSC populations differ significantly based on the donor’s genotype, source of isolation, and even a particular niche within the source tissue, these properties remain comparatively stable once the MSCs are characterized and expanded into a cell culture until at least passage 4–5 [351], making them seemingly more predictable as a result. This gives MSC-based models potential utility if both directed differentiation and a high degree of repeatability are desired at the same time.

While heterogeneity in iPSCs and MSCs cannot be avoided entirely, certain steps may still be taken to at least diminish it. Much thought has already been put into designing quality control and regulatory frameworks, as well as standard operation practices for iPSC use [352,353]. Nonetheless, iPSC heterogeneity cannot, as of yet, be eliminated and remains a major limiting factor in their use. Furthermore, new sources of heterogeneity are still being discovered. For example, after many conflicting reports, it has recently been confirmed that iPSCs generated from female donors do, after all, undergo the erosion of X chromosome inactivation, independent of global methylation levels or genomic imprinting, resulting in the reactivation of individual X-linked genes. This inactivation erosion was shown to be both highly variable and persistent throughout the differentiation process [354].

Regulations and reporting standards of MSC-based protocols are, in contrast, comparatively lacking; both in vitro and clinical studies rarely report on the exact marker landscape of MSCs, even at the level of minimum ICST criteria, with the reports on the culture conditions being similarly inconsistent [90,355]. This lack of standardization is incredibly concerning; different laboratories can end up producing MSC cultures with different properties from the exact same starting material because of variations in cell culture technique and reagents [356]. Factors such as the isolation method, composition of the expansion culture medium, and various environmental conditions all must be taken into account when designing an experiment with MSCs, and while all of them have already been extensively characterized [357], the development of exact regulations and widely acknowledged good laboratory and manufacturing practices still remains an ongoing process [358].

A potential way to navigate the heterogeneous MSC landscape would be to introduce additional markers into the analysis pipelines. Aside from the major MSC markers, which merely confirm the identity of the cells, new markers that denote the more qualitative properties of MSCs can be used to potentially identify the most promising MSC subpopulations within the heterogeneous populations. Two groups of such markers have been identified: the stemness markers, such as the SSEA-3, SSEA-4, NGFR, CD49F, GD2, CD349, Sca-1, and CD133, and the markers of specialized MSC populations, such as CD146, Nestin, CD200, CD106, CD142, and CD317, as reviewed in [359]. For example, CD146+ MSCs were demonstrated to have improved proliferative and differentiation capacity [360,361]. CD146 is assumed to modulate the differentiation potential of the cells through crosstalk with CD49f, which together regulate the lineage of MSCs presumably through interactions with key transcription factors, namely RUNX2, PPARγ, and C/EBPs [362]. Significantly improved immunomodulatory capabilities are also observed in CD146+ MSCs [363], notably through the elevated secretion of IL-6 and IL-8 [364,365] and improved Th17 cell suppression [366].

Another aspect of stem cell biology that remains relatively underappreciated within the context of in vitro disease modeling is the ability to modulate the properties of stem cells through their interactions with the underlying scaffolds. Substrate nanotopography is thought to affect the cells by interacting with the cell adhesion machinery, namely the focal adhesions. Nanotopography, similar in size and properties to integrin ligands, is able to bind the integrins, resulting in the contraction of the attached cytoskeleton [367]. This alone is enough to induce certain differentiation pathways in MSCs. Specific, not highly ordered, but not random either, patterning of the substrate can induce osteogenic differentiation purely through the action on focal adhesions. Whilst the effect is less pronounced compared to the conventional dexamethasone induction, with only 11 genes being upregulated compared to 24, the effect is nonetheless significant [368]. The effects of other types of substrate topologies on the cellular phenotype are widely reported and are summarized in [369].

The cause of this effect is thought to be the change in intracellular tension, which is known to impact the multipotent differentiation potential of cells through the YAP/TAZ pathway [370], translocation of actin into the nucleus [371], GPCRs and ROCK, and can be regulated through nanotopography by manipulating the interactions of integrins with cell adhesion machinery [372]; for example, through the enhancement of integrin endocytosis [373]. By reducing tension in MSCs through weakening their interactions with the patterned substrate (though not to a point when they begin to spontaneously differentiate in the adipogenic direction), their multipotent state can be made to persist in culture for longer durations. It is speculated that the reason for MSCs’ spontaneous in vitro differentiation is the unfamiliar mechanical properties of plastic cell culture vessels, which is seemingly the reason why placing cells on top of less stiff material can help delay this process [374]. Furthermore, these multipotency-extending materials can also be made with the ability to dynamically induce a specific differentiation pathway when needed. For example, these materials can be fabricated in such a way that after treatment with a peptidase (either user-added, such as elastases or secreted by cells endogenously under high confluence, such as certain matrix metalloproteinases in MSCs), a part of the matrix, marked by a preplaced enzyme-cleavable sequence, is removed, exposing a regulatory peptide to the cells. By presenting RGD (the cell adhesion-promoting peptide) to the MSCs in that way, osteogenic differentiation can be automatically induced once the cells have reached high confluence [375].

Nanoscale topography can be integrated into substrates with other large-scale surface structures. For example, in vitro osteogenesis is known to be supported by porous materials, with pores of different sizes having various roles. Large 100+ μm pores facilitate bone ingrowth and nutrient and waste transportation, while micropores of 0.5–10 μm promote cell adhesion and osteogenic differentiation. Combining multiple scales of pores within a single scaffold with additional nanotopographical patterning was shown to both enhance the osteogenic differentiation of rat BM-MSCs in vitro and result in improved bone regeneration after the scaffolds were transplanted at the sites of drill-induced bone damage in rats [376]. Nanotopography can also be combined with electrical stimulation—culturing stem cell-derived neurons on conductive substrates composed of layered assemblies of positively charged polymers and negatively charged carbon nanotubes both facilitate neural attachment through the formation of focal adhesions and allow tightly controlled electrical stimulation of the cells without the risk of culture medium electrolysis, which is associated with conventional metal electrodes. Such electrostimulation was previously shown to greatly enhance the neuronal differentiation capacity of neural stem cells, partially through the promotion of autophagy [377], and likely has the potential for iPSC and MSC manipulation, as well. Another intriguing approach is the integration of genetically engineered bacteria within the scaffolds, which can be designed to secrete specific factors capable of altering the properties of stem cells [378]. For example, integration of *L. lactis* engineered to secrete BMP-2 into the alginate hydrogel with BM-MSCs significantly enhances the efficiency of osteogenic differentiation of the latter: osteocalcin and osteopontin were found to be significantly elevated in cells cultured in the presence of BMP-2-secreting bacteria [379]. MSCs, in turn, can also be integrated into biomaterials as a secretory component, which is particularly prominent in neural regenerative strategies [380]. Embedding MSCs within electrospun fiber scaffolds, composed, for example, of reduced graphene oxide and polycaprolactone [381] or collagen with the addition of brain-derived neurotrophic factor [382], have been shown to result in improved MSC neuronal differentiation efficiency and were able to partially restore neural function in animal models of spinal cord injury.

The modulation of stem cell properties through substrate nanotopography is a rapidly developing field of study, and a comprehensive description of all recent advances is well beyond the scope of this text, having been extensively reviewed elsewhere [383]. Our aim was, rather, to highlight the potential of this approach, both for the in vitro modeling and for possible future therapeutic studies.

Finally, despite this review typically making a clear distinction between iPSC- and MSC-based in vitro models, it should be noted that in some cases, both types of cells are used to create a model. One particularly important effect is the stabilization of forming vasculature in MSC-EC co-culture [384], which can be exploited to enhance the vascularization of organoids [385]. Co-culture of iPSC-derived RPE, endothelial cells, and MSCs in a hydrogel-based ECM results in the formation of ROs that were able to closely replicate the tubular vascular networks characteristic of the choriocapillaris, with MSCs in particular contributing towards stabilizing them [149]. A similar effect has also been observed during the formation of cardiac organoids in co-culture with MSCs, where the latter helped stabilize the developing vessels through paracrine interactions [386].

In the context of disease modeling, it is often tempting to speculate that stem cell-based models might eventually supplant preclinical animal testing altogether. Indeed, as discussed in this review, iPSC- and MSC-based disease models are arguably more relevant for deciphering the mechanism of action of novel medicines compared to animal studies. They are cheaper and faster, can be used in high-throughput experiments, and do not have inter-species cross-reactivity limitations. Importantly, cell-based assays reduce animal use in accordance with the 3Rs principle [387]. However, these assays lack the complexity of tissues and organs; organoids and other emerging technologies should be used to increase the relevance of in vitro data [4]. Therefore, despite the continuous improvements in the biological accuracy of the models, in our opinion, this perspective remains fairly far-fetched at the time of writing this. Even state-of-the-art organ system-on-a-chip models are still unable to simulate the entire spectrum of cellular and molecular interactions that exist within the real tissues, while the cost efficiency of their use is not fully clear either, given the largely biased outlook on the problem that even the quantitative comparisons can show [388]. Therefore, complex stem cell-based models remain a predominantly academic endeavor, and until rigorous standardization pipelines are developed, it is likely to stay that way. Nonetheless, the FDA Modernization Act 2.0, signed into law in December 2022, ended the requirement of obligatory animal testing of novel medicines [389]. It supports the use of cell-based, computational, and other approaches instead of in vivo animal testing. However, further regulatory guidelines are necessary to define the requirements for cell-based disease models that guarantee their acceptance as reliable substitutes for in vivo animal studies.

iPSCs and MSCs were successfully used in off-the-shelf cell therapies, as discussed in this review. However, the extent of ex vivo manipulation in many of these products poses significant risks and regulatory concerns. It is illustrated by the complex regulatory history of the first FDA-approved MSC-derived cell therapy, remestemcel-L. It was repeatedly rejected due to concerns about clinical trial designs, potency assays, and manufacturing consistency [390]. Further studies are required to understand and reduce the tumorigenicity risks associated with MSC- and, especially, iPSC-based products. Harmonized guidelines on the assessment of tumorigenicity risks and the development of risk mitigation strategies will be of great value to the field. Platform bioprocess and platform analytical method developments may be key to cell therapies that are safe and economically feasible.

The use of iPSCs and MSCs is associated with far fewer ethical and legal concerns compared to ESC-derived cell therapies. However, attention should be paid to the issue of ownership of iPSC cell lines in case of their use in allogeneic cell products. An appropriate donor’s consent for their commercial use and for unknown future use has to be obtained. Donor consent should be compliant with the General Data Protection Regulation (GDPR) in the EU or the Health Insurance Portability and Accountability Act (HIPAA) in the US to protect the donor’s data. Data must be anonymized to prevent donor identification. This should prevent legal disputes similar to the litigation related to the HeLa cell line [391]. Harmonized requirements for the use of genetic information and the export of biological samples would facilitate the development and commercialization of off-the-shelf cell therapies.

Another major concern in regard to stem cells is the safety of their therapeutic use. The most pressing issues are the potential genomic instability of iPSCs [392] and their potential for malignant transformation. Relatively few studies have thus far assessed the risk of tumorigenesis associated with the therapeutic use of iPSCs. For example, it has previously been assessed [393] that iPSC therapy of a spinal cord injury in a murine model initially resulted in functional recovery but was eventually followed up by a delayed oncogenic transformation with an epithelial–mesenchymal transition. Such results are a major cause of concern and necessitate close, long-term observations of patients both during and after the clinical trials. Another major safety concern is the emergence of uncertified and unproven iPSC and MSC therapeutic approaches and their overuse by individual unscrupulous private clinics [394,395]. Addressing this issue requires both the education of the wider public on the risks and limitations of cell therapy in general, as well as the standardization of stem cell therapy product production pipelines, extensions of the scope of safety testing, and the unification of legal regulations on stem cell therapy across different countries [395,396].

One possible approach toward the standardization of iPSCs is the creation of allogeneic cell banks [397]. However, the use of both allogenic and even autologous iPSCs is associated with the risk of inflammation, a key aspect of their safety of use in cell therapy [398,399]. iPSC haplobanks can be utilized to reduce immune rejection [400] in addition to the more traditional approaches, such as the ones used to prevent graft-versus-host disease. These strategies include knockouts of the MHC-I, MHC-II, and B2M/HLA-E and the utilization of the immunosuppressors PD-L1, CD200, and CD47. Another effective approach involves the co-transplantation of iPSCs with other cells, which protects the former from the host immune response. Sertoli cells, MSCs, and regulatory T cells have all been used in this role [399]. The utilization of these strategies can help promote the immune tolerance of allogeneic iPSCs when they are used in a therapeutic context.

Despite the many caveats to their use, both iPSCs and MSCs have applications that they each excel at, which is what we sought to illustrate with this review. While obviously not a be-all-end-all instrument for uncovering the pathogenesis of various diseases and finding cures for them simultaneously, which is what the stem cells were often envisioned to be initially [401,402], mirroring similar overly optimistic expectations for finding a methodological panacea of sorts in the form of microarray technology [403], full genome sequencing [404], CRISPR/Cas9 [405], and, nowadays, big data and artificial intelligence [406,407], stem cells have nonetheless become an indispensable part of a cell biologist’s toolbox since the inception of this research field more than 20 years ago. Continued advances in the acquisition and characterization of different types of stem cells, refinement of cell culture techniques, and development of new, ever more elaborate stem cell-based in vitro models and disease treatment strategies all play a big part in the ongoing refinement of their application, with no end to it seemingly in sight.

## Figures and Tables

**Table 1:** Summary of clinical trials mentioned in the review.

	ClinicalTrials.gov ID	Title	Cell Type	Status	Conditions	Study Types	Phase
Inherited retinal diseases	NCT06891885	A Study to Investigate the Safety of DSP-3077 After a Unilateral Eye Injection in Male and Female Participants 18 Years of Age or Older with RP	iPSC	Not yet recruiting	RP	Interventional	Phase 1 Phase 2
	NCT06789445	A Study to Investigate the Safety of OpCT-001 in Adults Who Have Primary Photoreceptor Disease (CLARICO)	iPSC	Completed	Primary Photoreceptor Disease RP (RP) US	Interventional	Phase 1 Phase 2
	NCT01432847	Cell Collection to Study Eye Diseases	iPSC	Recruiting	Retinal Disease AMD Retinal Degeneration	Observational	*n/a
	NCT05800301	Management of RP Via Combination of Wharton’s Jelly-derived Mesenchymal Stem Cells and Magnovision	MSC	Completed	RP	Interventional	Phase 3
	NCT01531348	Intravitreal Injection of MSCs in RP	MSC	Completed	RP	Interventional	Phase 1
	NCT05909488	Role of UC-MSC and CM to Inhibit Vision Loss in RP Phase I/II	MSC	Recruiting	RP	Interventional	Phase 2 Phase 3
	NCT03011541	Stem Cell Ophthalmology Treatment Study II (SCOTS2)	MSC	Recruiting	Retinal Disease ARD RP	Interventional	n/a
NDevDs & NDDs	NCT03635294	Multi-Omics and IPSCs to Improve Diagnosis of Rare Intellectual Disabilities	iPSC	Completed	Rare Intellectual Disabilities	Interventional	n/a
	NCT02980302	Development of the Tool “iPSC” for the Functional Study of Mutations Responsible for Mental Retardation	iPSC	Completed	Intellectual Deficiency	Interventional	n/a
	NCT02720939	ASD-specific Induced Pluripotent Stem Cells for Disease Modeling	iPSC	Completed	ASD	Observational	n/a
	NCT06821529	Stereotactic Intracerebral Injection of IPSC-DAPs in Patients with Parkinson’s Disease	iPSC	Not yet recruiting	PD	Interventional	Phase 1
	NCT06778265	An Exploratory Clinical Study of UX-DA001 in Subjects with Idiopathic Parkinson’s Disease	iPSC	Enrolling by invitation	PD, Idiopathic	Interventional	Phase 1
	NCT06765564	Clinical Study of Induced Pluripotent Stem Cells Derived Motor Neuron Precursor Cell Therapy for Amyotrophic Lateral Sclerosis (ALS)	iPSC	Recruiting	ALS	Interventional	n/a
	NCT06687837	Treating Parkinson’s Disease Through Transplantation of Autologous Stem Cell-Derived Dopaminergic Neurons	iPSC	Recruiting	PD	Interventional	Phase 1
	NCT06482268	Transplantation of Human iPS Cell-derived Dopaminergic Progenitors (CT1-DAP001) for Parkinson’s Disease (Phase I/II)	iPSC	Recruiting	PD	Interventional	Phase 1
	NCT06422208	Autologous iPSC-Derived Dopamine Neuron Transplantation for Parkinson’s Disease	iPSC	Enrolling by invitation	PD	Interventional	Phase 1
	NCT06344026	Phase 1/2a Study of ANPD001 in Parkinson’s Disease	iPSC	Enrolling by invitation	PD	Interventional	Phase 1
	NCT06145711	A Clinical Trial of Parkinson’s Disease Treatment by HiPSCs-Derived Dopaminergic Neural Precursor Cells	iPSC	Recruiting	PD	Interventional	n/a
	NCT03322306	Establishment of Genetic Basis for Neurological Disease by Genetic Screening	iPSC	Enrolling by invitation	Neurodegenerative Disease	Observational	n/a
	NCT00874783	Development of IPS from Donated Somatic Cells of Patients with Neurological Diseases	iPSC	Recruiting	Neurodegenerative Disorders	Observational	n/a
	NCT03550183	Umbilical Cord-Derived Mesenchymal Stem Cells Therapy in Parkinson’s Disease	MSC	Unknown status	PD	Interventional	Phase 1
	NCT04821479	Repeated Mesenchymal Stem Cell Injections in ALS	MSC	Completed	ALS	Interventional	Phase 1 Phase 2
	NCT06910384	A Study of STRO4 in Patients with Amyotrophic Lateral Sclerosis (ALS)	MSC	Not yet recruiting	ALS	Interventional	Phase 2
Skin	NCT02579369	Study to Evaluate the Safety of ALLO-ASC-DFU in Subjects with Dystrophic Epidermolysis Bullosa	MSC	Completed	Dystrophic EB	Interventional	Phase 1 Phase 2
	NCT03529877	Allogeneic ABCB5-positive Stem Cells for Treatment of Epidermolysis Bullosa	MSC	Completed	Recessive Dystrophic EB	Interventional	Phase 1 Phase 2
Hemophilia	NCT02108132	Allogenic Bone Marrow-Derived Mesenchymal Stem Cell Therapy in Cases of Hemophilia	MSC	Unknown status	Hemophilia	Interventional	Phase 1
	NCT05187936	Preclinical Models for Mesenchymal Stem Cell Therapy in Hemophilic Arthropathy	MSC	Unknown status	Hemophilia A Hemophilia B Arthropathy	Observational	n/a
Marfan Syndrome	NCT02815072	Generation of Marfan Syndrome and Fontan Cardiovascular Models Using Patient-specific Induced Pluripotent Stem Cells	iPSC	Unknown status	MS	Observational	n/a
Cystic Fibrosis	NCT03754088	In Vitro Model of the Cystic Fibrosis Bronchial Epithelium Via iPS Technology	iPSC	Completed	CF	Observational	n/a
	NCT02866721	Safety and Tolerability Study of Allogeneic Mesenchymal Stem Cell Infusion in Adults with Cystic Fibrosis	MSC	Completed with results	CF	Interventional	Phase 1
	NCT03058068	Human Mesenchymal Stem Cells Infusion in Patients with Cystic Fibrosis	MSC	Withdrawn	CF	Interventional	Phase 1
Muscular dystrophies	NCT02413450	Derivation of Human-Induced Pluripotent Stem (iPS) Cells to Heritable Cardiac Arrhythmias	iPSC	Enrolling by invitation	Inherited Cardiac Arrhythmias Long QT Syndrome (LQTS) Brugada Syndrome (BrS)	Observational	n/a
	NCT02285673	Efficacy of Umbilical Cord Mesenchymal Stem Cells in Duchenne Muscular Dystrophy	MSC	Unknown status	DMD	Interventional	Phase 1 Phase 2
	NCT02235844	Allogeneic Human Umbilical Cord Mesenchymal Stem Cells for a Single Male Patient with Duchenne Muscular Dystrophy (DMD)	MSC	Completed	DMD	Interventional	Phase 1
	NCT01610440	Safety and Efficacy of Umbilical Cord Mesenchymal Stem Cell Therapy for Patients with Duchenne Muscular Dystrophy	MSC	Unknown status	DMD	Interventional	Phase 1 Phase 2
	NCT06328725	Evaluate the Efficacy and Safety of EN001 in Patients with Duchenne Muscular Dystrophy	MSC	Not yet recruiting	DMD	Interventional	Phase 1 Phase 2

*n/a—not available.

## Abbreviations

The following abbreviations are used in this manuscript:

iPSC	Induced Pluripotent Stem Cell
MSC	Mesenchymal Stem Cell
3D	Three-Dimensional
2D	Two-Dimensional
ECM	Extracellular Matrix
ESC	Embryonic Stem Cells
PR	Progesterone Receptor
PEG	Poly Ethylene Glycol
ISCT	International Society for Cell & Gene Therapy
BM-MSC	Bone Marrow-Mesenchymal Stem Cell
UC-MSC	Umbilical Cord-Mesenchymal Stem Cell
WJ-MSC	Wharton Jelly-Mesenchymal Stem Cell
ASC	Adipose Stem Cell
DPSC	Dental Pulp Stem Cell
AM-MSC	Amniotic Membrane-Mesenchymal Stem Cell
IRD	Inherited Retinal Disease
RPE	Retinal Pigment Epithelium
RGC	Retinal Ganglion Cells
RP	Retinitis Pigmentosa
LCA	Leber Congenital Amaurosis
SD	Stargardt Disease
AMD	Age-related Macular Degeneration
AAV	Adeno-Associated Virus
OS	Outer Segments
RO	Retinal Organoid
cGMP	cyclic Guanosine Monophosphate
NDevD	Neurodevelopmental Disease
NDD	Neurodegenerative Disease
RTT	Rett Syndrome
ASD	Autism Spectrum Disorders
AD	Alzheimer’s Disease
PD	Parkinson’s Disease
ALS	Amyotrophic Lateral Sclerosis
HD	Huntington’s disease
MenSC	Menstrual Stromal Cell
BO	Brain Organoid
BBB	Blood–Brain Barrier
NMJ	Neuromuscular Junction
ID	Intellectual Disability
DAP	Dopamine Progenitor
EB	Epidermolysis Bullosa
ARCI	Autosomal Recessive Congenital Ichthyosis
KLC	Keratocyte-like Cell
SkO	Skin Organoid
TTD	Trichothiodystrophy
EBS	Epidermolysis Bullosa Simplex
RDEB	Recessive Dystrophic Epidermolysis Bullosa
FVIII	Factor VIII
FIX	Factor IX
EF-1α	Elongation Factor-1 alpha
MFS	Marfan Syndrome
VSMC	Vascular Smooth Muscle Cell
SHF-VSMC	Second Heart Field-Vascular Smooth Muscle Cell
NC-VSMC	Neural Crest-Vascular Smooth Muscle Cell
MRC2	Mannose Receptor 2
EB	Embryoid Body
CF	Cystic Fibrosis
CFTR	Cystic Fibrosis Transmembrane conductance Regulator
FIS	Forskolin-Induced Swelling
MD	Muscular Dystrophy
UCMD	Ulrich Congenital Muscular Dystrophy
DMD	Duchenne Muscular Dystrophy
BMP	Bone Morphogenic Protein
FGF	Fibroblast Growth Factors
EDMD1	Emery–Dreifuss Muscular Dystrophy type 1
EFS	Electrical-Field Stimulation
ROS	Reactive Oxygen Species
XF-iMSC	Xeno-Free iPSC-derived Mesenchymal Stem Cell
DEC	Dystrophin-Expressing Chimeric (cells)
OI	Osteogenesis Imperfecta
ACH	Achondroplasia
UPR	Unfolded Protein Response
ER	Endoplasmic Reticulum
PKU	Phenylketonuria
GDPR	General Data Protection Regulation
HIPAA	Health Insurance Portability and Accountability Act
